# Halogenated imidazole derivatives block RNA polymerase II elongation along mitogen inducible genes

**DOI:** 10.1186/1471-2199-11-4

**Published:** 2010-01-15

**Authors:** Michal Mikula, Karolina Hanusek, Agnieszka Paziewska, Artur Dzwonek, Tymon Rubel, Karol Bomsztyk, Jerzy Ostrowski

**Affiliations:** 1 Department of Gastroenterology, Maria Sklodowska-Curie Memorial Cancer Center and Institute of Oncology, Warsaw, Poland; 2 Department of Gastroenterology and Hepatology, Medical Center for Postgraduate Education, Warsaw, Poland; 3 UW Medicine Lake Union, University of Washington, Seattle, USA

## Abstract

**Background:**

Aberrant activation of protein kinases is one of the essential oncogenic driving forces inherent to the process of tumorigenesis. The protein kinase CK2 plays an important role in diverse biological processes, including cell growth and proliferation as well as in the governing and transduction of prosurvival signals. Increased expression of CK2 is a hallmark of some cancers, hence its antiapoptotic properties may be relevant to cancer onset. Thus, the designing and synthesis of the CK2 inhibitors has become an important pursuit in the search for cancer therapies.

**Results:**

Using a high-throughput microarray approach, we demonstrate that two potent inhibitors of CK2, 4,5,6,7-tetrabromo-benzimidazole (TBBz) and 2-Dimethyloamino-4,5,6,7-tetrabromo-1H-benzimidazole (DMAT), blocked mitogen induced mRNA expression of immediate early genes. Given the impact of these inhibitors on the process of transcription, we investigated their effects on RNA Polymerase II (RNAPII) elongation along the mitogen inducible gene, *EGR1 *(early growth response 1), using chromatin immunoprecipitation (ChIP) assay. ChIP analysis demonstrated that both drugs arrest RNAPII elongation. Finally, we show that CDK9 kinase activity, essential for the triggering of RNAPII elongation, was blocked by TBBz and to lesser degree by DMAT.

**Conclusions:**

Our approach revealed that small molecules derived from halogenated imidazole compounds may decrease cell proliferation, in part, by inhibiting pathways that regulate transcription elongation.

## Background

Phosphorylation is the most common post-translational protein modification that regulates a wide spectrum of cellular processes [1]. Protein kinases modify the targeted protein by transferring phosphate groups from ATP or GTP to free hydroxyl groups of serine, threonine or tyrosine in protein amino acid backbone causing conformational change in the protein structure. It has been estimated that approximately one-third of the eukaryotic proteome is phosphorylated at any given time. Dysregulation of protein kinase-mediated signaling pathways may impair cell growth, proliferation and apoptosis, leading to various disease states [2]. The success of the kinase inhibitor imatinib mesylate (Gleevec) in treatment of selected cancers has generated great interest and hope to use inhibitors of this class of enzymes to treat cancer including promising results with the use of CK2 small molecule inhibitors [3-5].

Specificity of phosphorylation by protein kinases is important for the fidelity of signal transduction largely determined by amino acids flanking Ser/Thr/Tyr residues and kinase-substrate concentrations *in situ *[6]. The constitutively active CK2 kinase is the most pleiotropic protein kinase known; it phosphorylates multiple cellular proteins both *in vitro *and *in vivo *[3]. CK2 is required for cell viability and it is involved in regulation of almost all stages of the cell cycle in yeast and mammals [7-12]. Increased expression of CK2 is one of the hallmarks of cancers including the lung, mammary gland, kidney and prostate [3]. This observation has generated great interest and has fueled the search for specific inhibitors of this enzyme.

The ATP analog 5,6-dichloro-1-b-D-ribofuranosylbenzimidazole (DRB) was one of the earliest CK2 inhibitors used. Modifications of the DRB structure by removing the sugar moiety and replacing the chlorines with bromine atoms produced the 4,5,6,7-tetrabromo-1H-benzotriazole, TBB. Further reactions within triazole ring generated 4,5,6,7-tetrabromo-benzimidazole (TBBz) and 2-Dimethyloamino-4,5,6,7-tetrabromo-1H-benzimidazole (DMAT). Both compounds were shown to be potent CK2 inhibitors, *in vitro *[13]. Although CK2 inhibitors exhibit different efficacy and specificity, almost all of them inhibit cell proliferation and induce caspase-related apoptosis in the established cancer cell lines [3]. Here, we used several assays to examine the mode of action of TBBz and DMAT *in vivo*.

## Results

### Inhibition of cell proliferation by TBBz and DMAT in HeLa cells

**Figure 1:**
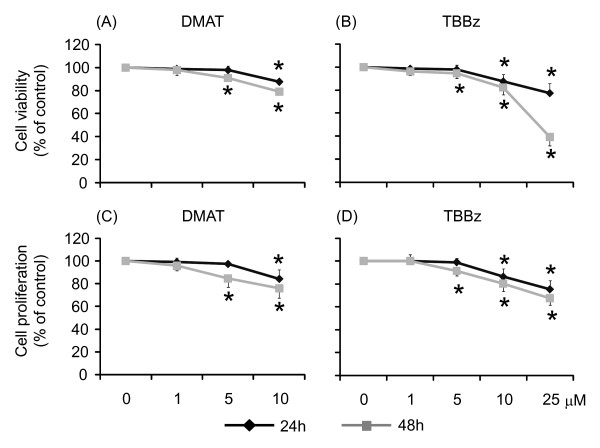
**The inhibitory effects of DMAT and TBBz on viability and proliferation of HeLa cells**. Cells were grown in the presence of 1, 5 and 10 μM of DMAT (A, C) or 1, 5, 10 and 25 μM of TBBz (B, D). Cell viability was monitored by MTT test (A, B), and cell proliferation by ^3^H thymidine incorporation (C, D) 24 and 48 h later. Four independent experiments were performed, and all assays were repeated in octuplicate. Results are expressed as the percentage of control cell viability or proliferation and represent means ± S.D. *; P < 0.05 compared to the control.

The reduction of tetrazolium salts to formazans by living cells results in the color development in the MTT test and reflects the combined effects of cell proliferation and survival. HeLa cells were treated with increasing concentrations of TBBz or DMAT and MTT test was performed after 24 and 48 h of the treatment. The suppressive effect of both CK2 inhibitors on cell growth was observed with the highest concentration of inhibitors; 10 μM of DMAT and 25 μM of TBBz (Figure 1A, B). The results of the MTT test were further confirmed by [^3^H] thymidine incorporation assays. Again, the proliferation of HeLa cells was inhibited after 24 h (and to a higher degree after 48 h) of treatment with 10 and 25 μM of DMAT and TBBz, respectively (Figure 1C, D). The observed inhibitory effect of both TBBz and DMAT on cell proliferation is in agreement with previously published results by Pagano et al. [14], however the inhibition efficacy varies considerably between Jurkat cells used in that study and the HeLa cells used here (viability 25% and 85% respectively, Figure 1A).

### TBBz and DMAT effects on gene expression profiles

**Figure 2:**
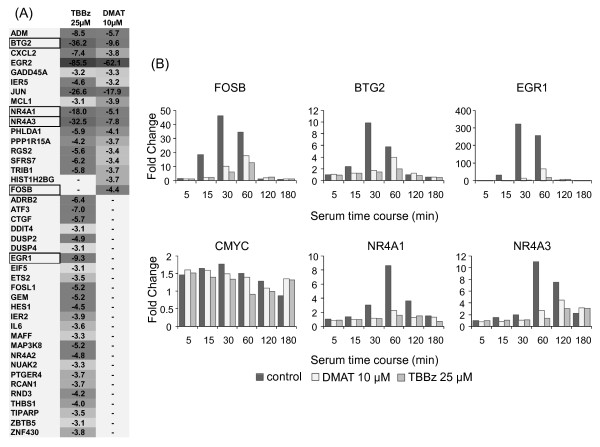
**Changes in gene expression induced by TBBz and DMAT**. (A) List of 42 genes and fold change in their expression in response to treatment with inhibitors (1 hr) were analyzed by the Affymetrix U133A 2.0 GeneChip microarray. Dark grey indicates a high decrease and light grey lower decrease in expression. Expression of the boxed genes was confirmed by real time PCR and is presented as fold change at zero time (B).

To explore the molecular mechanism of action of these inhibitors in more detail, we performed an oligonucleotide microarray experiment. We hybridized cRNA prepared from RNA of control HeLa cells and cells treated for 1, 6 and 24 hours with either 25 μM TBBz or 10 μM DMAT to Affymetrix U133A 2.0 GeneChip oligo-microarrays containing 22277 probe sets mapping to 14500 well-characterized human genes. In these studies, we established that treatment of serum-starved quiescent cells with 15% of fetal bovine serum changed the level of 925 out of 10,600 transcripts (more than 2-fold change; FDR = 0.001) at least at one of the analyzed time points [Additional file 1: Table S1]. Inhibitor treatment for 1 and 6 hrs mainly caused a decrease in transcript levels (Table 1), and most of the changes at 1 hr of treatment were observed among immediately-early genes (Figure 2A).

**Table 1:** Number of probe sets/genes predominantly affected by CK2 inhibitors.

		Differentially expressed		Underexpressed		Overexpressed	
		
Inhibitor	Time point (hr)	Probe sets	Genes	Probe sets	Genes	Probe sets	Genes
TBBz	1	65	51	63	49	2	2
	6	818	692	742	624	77	70
	24	319	272	162	142	157	130

DMAT	1	29	23	28	22	1	1
	6	223	192	185	162	38	30
	24	66	57	27	22	39	35

To confirm microarray data, next we examined the effect of the inhibitors on serum-induced transcription of the selected immediate early genes, *EGR1 *(early growth response-1), *FOSB *(FBJ murine osteosarcoma viral oncogene homolog B), *BTG2 *(B-cell translocation gene 2), *NR4A1 *(nuclear receptor subfamily 4, group A, member 1), *NR4A3 *( nuclear receptor subfamily 4, group A, member 3) and *CMYC *(v-myc myelocytomatosis viral oncogene homolog) [15], by RT qPCR. As shown in Figure 2B, serum stimulation of HeLa cells was accompanied by rapid induction of all transcripts, except *CMYC*, with a peak increase at 30-60 min of stimulation followed by a continual decline to basal expression levels at 180 min, the latest experimental time point. The highest increase was found for *EGR1 *mRNA (~300 fold change), while *CMYC *transcript increased less than two-fold. Inhibitor treatment significantly decreased the transcript levels at almost all time points. These results showed an inhibitory effect of both drugs on the induced transcription of immediate early genes suggesting that both agents may inhibit RNA Polymerase II (RNAPII) elongation. This possibility was tested next.

### TBBz and DMAT inhibit the elongation phase of transcription

Transcription complexes of RNAPII often pause near the transcription start site (TSS) [16], and the transition to elongation depends on phosphorylation of the carboxy-terminal domain (CTD) of the largest subunit of RNAPII. The CTD consists of multiple heptapeptide repeats with the consensus amino acid sequence (YSPTSPS) and phosphorylation on Ser-2 and Ser-5 residues is mediated by the homologous cyclin dependent kinases (CDK), CDK7, -8 and -9, ERK-1/2, and c-ABL [17].

A potent inhibitor of CDK9, DRB behaves as global inhibitor of transcriptionally inducible genes [18]. Both TBBz and DMAT belong to the class of halogenated imidazole products whose structure was derived from DRB.

**Figure 3:**
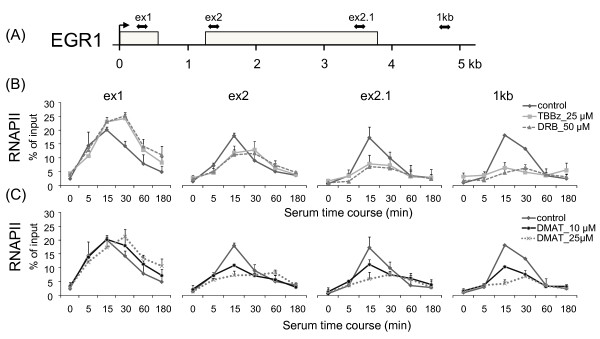
**TBBz and DMAT arrest RNAPII elongation along the *EGR1 *locus after serum treatment**. (A) Position of primers relative to transcription start sites (in bp): exon 1 +248; exon 2 +1287; exon 2.1 +3490; 1 kb +4528. (B, C) HeLa cells maintained for 48 h in 0.5% serum were treated with fresh medium supplemented with 15% FBS and -/+ 25 μM TBBz/50 μM DRB/10 or 25 μM DMAT (C) for the indicated times and then used in ChIP assays with antibodies to RNAPII. Purified DNA was used in real-time PCR with pairs of primers spanning the *EGR1 *locus. The density of RNAPII on the *EGR1 *gene was quantified by real-time PCR and is presented as a percentage of input. Data represent means ± S.D from 3 independent experiments.

**Figure 4:**
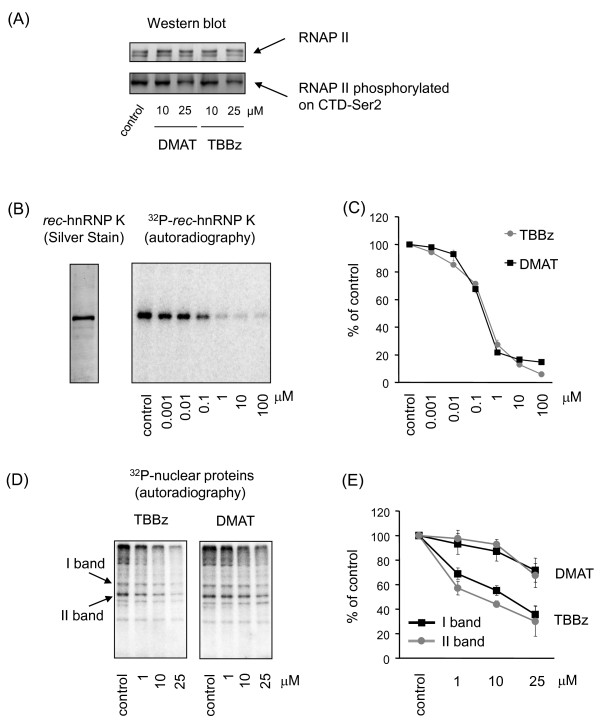
**The effects of DMAT and TBBz on phosphorylation of RNAPII and other nuclear proteins**. (A) Equal amounts of nuclear extracts prepared from untreated and inhibitor-treated cells (60 min) were separated by SDS-PAGE followed by Western blotting with anti-RNA PII CDT repeat (ab5408, Abcam) or anti-Ser2-phospho-RNAPII CDT (ab5095, Abcam). Similar patterns were obtained in 3 different experiments using different nuclear extracts. (B) Bacterially expressed recombinant hnRNP K protein (rec-hnRNPK, left panel) was phosphorylated by CK2 kinase, purified from rat liver (Sigma; C3460), with increasing concentrations (0; 0.001; 0.01; 0.1; 1.0, 10; 100 μM) of TBBz or DMAT. Assays were stopped by boiling samples in Laemmli loading buffer. K protein was separated by SDS-PAGE, and dried gels were autographed (right panel - representative gel with TBBz treatment). (C) Densitometry of the results with inhibitors are expressed as the percentage of control kinase activity and represent means ± S.D from 3 independent experiments. (D) Nuclear extracts prepared from untreated and inhibitor-treated cells were used for autophosphorylation reactions with or without 1, 10, and 25 μM TBBz or DMAT. Assays were stopped by boiling samples in Laemmli loading buffer. Proteins were separated by SDS-PAGE, and dried gels were autoradiographed (Phosphorimager) (E). Densitometrical analysis of two phosphorylated protein bands [marked on panel (D)] is shown as means ± S.D from results expressed as the percentage of controls.

To establish whether TBBz and DMAT arrest elongation phase of transcription, the kinetics of RNAPII binding within the *EGR1 *gene was further studied using the Matrix-ChIP assay. RNAPII occupancy was assayed at 4 positions along the *EGR1 *gene, at exon1 (ex1) and the beginning (ex2) and end (ex2.1) of exon2, as well as at a site 1 kb downstream of the poly-adenylation (A) signal (Figure 3A). The latter site was chosen because it was shown for several genes that the RNAPII complex continues elongation beyond the poly-(A) site and pauses within 0.5 - 1.5 kb downstream. This process plays an important role in the maturation of the nascent transcript [19]. The ChIP results show that both TBBz and DMAT caused an increase in RNAPII occupancy at the first exon of *EGR1 *compared to control (no inhibitors) but suppressed inducible elongation of RNAPII at all regions further downstream after 30 min of serum stimulation (Figure 3B, C). DRB exhibited similar inhibitory effects. This suggested that the inhibitors were preventing RNAPII elongation but not recruitment to the *EGR1 *promoter. Because both agents inhibited transcription elongation, and both are derivatives of DRB, we reasoned that they might affect CDK9 kinase activity. According to the postulated model, transcription initiation of RNAPII is regulated by CDK7/CDK8-mediated phosphorylation of CTD at Ser-5 near the TSS. Further down-stream phosphorylation of the CTD at Ser-2 is required for RNAPII to transition to elongation phase [16]. Ser2 phosphorylation is mediated by CDK9, a kinase that exist in a complex with cyclin T in the positive elongation factor b, P-TEFb. Western blot analysis revealed that both TBBz and DMAT at a concentration 25 μM decreased phosphorylation of the CTD at Ser-2 residues (Figure 4A, lower panel) while the levels of CTD RNAPII were not changed (Figure 4A, upper panel) suggesting inhibition of CDK9 kinase activity.

### TBBz and DMAT inhibit the activity of CDK9

To test the effect of CK2 inhibitors on CDK9 activity, several *in vitro *phosphorylation assays were performed. Both TBBz and DMAT produced the same dose-dependent decrease in *in vitro *phosphorylation of hnRNP K protein, a well characterized CK2 substrate [20] (Figure 4B, C). They also inhibited the autophosphorylation of several protein bands when nuclear extracts (NE) were used as a source of both kinase activity and substrate proteins (Figure 4D). Densitometric analysis of two highly phosphorylated protein bands revealed that both inhibitors decreased the level of their phosphorylation; TBBz, however, inhibited phosphorylation more efficiently than DMAT (Figure 4E).

**Figure 5:**
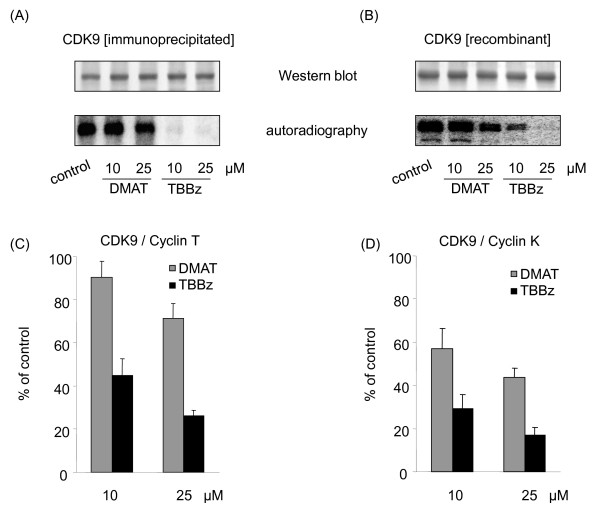
**Differential inhibitory effects of DMAT and TBBz on CDK9 activity *in vitro***. (A) CDK9/Cyclin T1 proteins pulled down from nuclear extracts (anti-CDK9+anti-cyclin T1, Santa Cruz Biotechnology, D-7 and H-245, respectively) were used in CDK9 autophosphorylation reactions without or with DMAT or TBBz. Autophosphorylated proteins were resolved by SDS-PAGE, transferred to PVDF membrane and immunostained by anti-CDK9 antibody (upper panels) and scanned using a Phosphorimager (lower panels). Similar patterns were obtained in 3 different experiments. (B) Recombinant fusion full-length human CDK9+CyclinK proteins, co-expressed by baculovirus in Sf9 insect cells (Abcam, ab70320) were used in autophosphorylation assays as above. (C) Complexes of CDK9/Cyclin T1 proteins pulled down from nuclear extracts or (D) human CDK9+CyclinK protein were used in kinase assays, with or without DMAT or TBBz. The heptapeptide YSPTSPS was used as a substrate. The reaction mixtures were applied to acidic hydrolysis of γ^32^P- ATP followed by phosphomolybdate extraction, and ^32^P-phosphopeptide was determined by liquid scintillation spectrophotometry. Results are expressed as a percentage of kinase inhibition and represent means ± S.D. of 2 separate experiments.

To test the effect of CK2 inhibitors on CDK9 activity, NE proteins were *in vitro *phosphorylated with or without inhibitors and then were immunopecipitated with antibodies to both CDK9 and its partner cyclin T1. TBBz but not DMAT inhibited phosphorylation of precipitated protein with the molecular weight around 40 KDa, which corresponds to molecular weight of CDK9. These data suggested that TBBz targets components of the P-TEFb complex (Figure 5A). The CDK9/cyclin-K complex has also a kinase activity towards the CTD domain of RNAP II and can substitute P-TEFb *in vitro *[21]. Thus, we tested the specificity of the drugs inhibitory effect on CDK9 autophosphorylation using a recombinant fusion full-length human CDK9-Cyclin K protein (Figure 5B). Treatment with 10 μM TBBz significantly decreased CDK9 phosphorylation while the 25 μM concentration completely inhibited it (Figure 5B, lower panel). On the other hand, DMAT at concentration of 10 μM had little or no effect on CDK9 autophosphorylation and at higher concentration (25 μM) there was a decrease in signal which was less pronounced than that for 10 μM TBBz.

To confirm the inhibitory effect of TBBz and DMAT on CDK9-mediated phosphorylation of CDK, we carried out additional experiments. Proteins immunoprecipitated (anti-CDK9+anti-cyclin T1 antibodies) from nuclear extracts (Figure 5C) and CDK9-Cyclin K recombinant fusion protein complex (Figure 5D) were tested in kinase assays using CTD heptapeptide repeats (YSPTSPS) as a substrate. Both assays revealed that TBBz and DMAT inhibited kinase activity of CDK9, although the latter agent was a less effective inhibitor.

## Discussion

Induction of cell proliferation is associated with transcriptional stimulation of growth-related genes that are required for the G_1_/S transition [22]. The mitogenic stimulation of various cell types is accompanied by rapid induction of immediate-early genes [23]. These genes are involved in cell proliferation, differentiation, apoptosis, and oncogenic transformation [24]. They regulate G_1_/S transition in the cell cycle and represent diverse functional classes of proteins including transcription factors. These processes are controlled by external stimuli [25,26] which activate kinase cascades that transduce mitogenic signals to the nucleus [27]. Protein kinases are involved in the regulation of most cellular functions, including cell cycle control, proliferation, and differentiation [28,29]. CK2 is one of the most pleiotropic protein kinases and displays constitutive catalytic activity [30,31]; it promotes cell survival and plays an anti-apoptotic role [3,32].

Halogenated imidazole class of small molecules are chemotherapeutic candidates but the mode of their action remains poorly understood [3]. In the present study we examined the molecular mechanisms that account for the anti-proliferative activity of two members of this class of drugs, TBBz and DMAT. Using microarray analysis we demonstrated that both inhibitors impaired expression of serum induced immediate-early genes, suggesting that these inhibitors block mitogenic signaling cascades. Next, using the Matrix-ChIP assay we found that both compounds inhibited transcription elongation similarly to the known transcription inhibitor, DRB, by arresting RNAPII near the TSS.

Although both TBBz and DMAT are potent inhibitors, none of the halogenated benzimidazoles affect selectively CK2 kinase activity. In fact, only quinalizarin (1,2,5,8-tetrahydroxyanthraquinone) which belongs to the same chemical class as emodin, has been shown to be the most potent and selective CK2 inhibitor characterized so far, as recently reported by Cozza et al. [33]. However, the specificity of CK2 inhibitors action *in vivo *has not been fully characterized. Therefore, our studies may provide a better understanding of the off-target effects of CK2 inhibitors. The routinely used *in vitro *assays test specificity of CK2 inhibitors on a limited set of kinases do not reflect the physiological conditions where other kinases can be directly or indirectly affected by the drugs.

Given the pro-survival role of elevated CK2 expression in cancer cell lines, a number of studies have been done to determine the effect of CK2 inhibitors and their value as anti-cancer agents [3]. A number of cell lines treated with CK2 inhibitors responded with the induction of apoptosis through the activation of caspases. However, the extent of this process and the dose of inhibitor used differed between the cell lines. With respect to the inhibitors investigated in our study, the viability of HeLa cells was reduced to 85% after 24 hours treatment with DMAT, while in another study at the same time point, Jurkat cell viability dropped to 25% [14]. Contrary to the results with DMAT, Jurkat cells responded similarly to HeLa cells when treated with TBBz, with viability reduced to 75% after 24 h incubation. HL-60 cells, however, were more potently affected as their viability was decreased to 30% by TBBz treatment [34].

Gene transcription in eukaryotes is carried out by the three different DNA dependent RNA polymerases RNAPI, RNAPII, and RNAPIII. With regard to the process of transcription, CK2 is involved in the regulation of transcription driven by RNAPI and RNAPIII. CK2 was found at the rDNA promoter where it interacts with RNAPIβ and phosphorylates several components of the RNAPI transcription complex [35,36]. CK2 also plays a fundamental role in the regulation of RNAPIII transcription [37] as it binds to the RNAPIII complex associated with the U6 promoter, and, through phosphorylation of RNAPIII associated proteins, plays both positive and negative regulatory roles in the transcription driven by this polymerase [38]. To date, there is less evidence supporting a role of CK2 in the regulation of RNAPII transcription as compared to the other two polymerases. It has been shown that phosphorylation of TFIIA, TFIIE, TFIIF (General Transcription Factors) and RNAPII by CK2 can modify the formation of transcription complexes on the Ad-MLP promoter [39]. CK2 also phosphorylates FCP1 (TFIIF-dependent CTD phosphatase I) one of the major phosphatases known to dephosphorylate the RNAPII CTD [40]. CK2-dependent phosphorylation greatly enhances the CTD phosphatase activity of FCP1 and FCP1-dependent dephosphorylation of the CTD domain is essential for recycling of RNAPII and transcription reinitiation [40,41]. Taken together, these observations present CK2 as a key factor in the regulation of transcription driven by three nuclear polymerases and we cannot exclude the possibility that the role of CK2 in the regulation of RNAPII transcription is far greater than currently known. So, it is possible that inhibitors used here impair immediate-early genes expression, in part, through CK2.

The inhibitors used in this study, TBBz and DMAT, were previously considered as specific to CK2 kinase. However, according to the results published by Pagano et al., both TBBz and DMAT appear to be also powerful inhibitors of member of three kinase subfamilies (PIM - provirus integration site for Moloney murine leukaemia virus, HIP - homeodomain-interacting protein, DYRK - dual specificity protein kinase), as well as of PKD1 (protein kinase D1) and CDK2 [42]. The spectrum of kinases affected by both inhibitors was similar for both compounds, however the inhibitory effect of DMAT on these kinases was slightly higher than that of TBBz. Interestingly, DMAT displayed a promiscuous nature inhibiting by > 50%, 34 kinases out of 70 tested in vitro [42]. This observation is not surprising since the catalytic sites of most human kinases are highly conserved, and cell-permeable kinase inhibitors are mostly competitive with ATP for their ATP-binding pocket. Thus, inhibitors may similarly target representatives of different branches of the kinome, and the final biological effect of these inhibitors likely reflects functions of the multiple kinases affected.

In eukaryotes, multiple steps of transcription are controlled by phosphorylation of the RNAPII CTD domain, mediated by CDKs. CDK7 and CDK8 regulate the processes involved in transcription initiation while CDK9 regulates elongation [16]. Recent evidence indicates that a large number of genes are regulated through promoter-proximal pausing (PPP) of RNAPII. Promoters of some genes with PPP were found to be occupied by the transcriptional machinery but did not produce transcripts, indicating that polymerase recruitment is not rate limiting for expression of those genes. PPP is particularly prevalent at genes involved in development and in the response to stimuli [43]. The RNAPII CTD is hypo-phosphorylated when initially engaged to the promoter, and undergoes serial phosphorylations at Ser5 during promoter clearance. This is followed by the binding of DRB sensitivity-inducing factor (DSIF) and negative elongation factor (NELF) which, without activity by CDK9, cause RNAPII to pause [16]. A classic inhibitor of transcription, DRB, which is also the first known inhibitor of CK2, arrests global RNAPII-dependent transcription by inhibiting CDK9, which normally phosphorylates the CTD at ser2 as well as DSIF and NELF [16]. In this case, inhibition of gene transcription by DRB may mostly reflect its inhibitory effect on CDK9 rather than CK2. In these studies, we demonstrate that TBBz, and at the lesser degree DMAT, block phosphorylation of CDK9 and its activity *in vitro *(Figure 5) and both inhibitors decrease CTD phosphorylation *in vivo *(Figure 4A), suggesting the means by which they inhibit RNAPII elongation. Knowing the promiscuous nature of the halogenated benzimidazoles it is possible that, apart from CDK9, TBBz and DMAT may inhibit also other kinases which activity is essential for RNAPII elongation.

## Conclusions

In sum, we used a combination of *in vivo *and *in vitro *approaches to evaluate the mode of action of two halogenated imidazole derivative TBBz and DMAT. Both agents inhibited cell proliferation and mRNA expression and transcription elongation but spectra of their molecular targets may not be the same. Our approach could be used for testing an increasing numbers small molecules derived from DMAT/TBB structure, for their affect on transcription elongation by using the Matrix-ChIP assay to asses RNAPII density at inducibly transcribed loci.

## Methods

### Cells

HeLa cells were grown in plastic cell culture flasks in DME media supplemented with 10% FBS, 2 mM glutamine, penicillin (100 units/ml), streptomycin (0.01%), and humidified with 6/94% CO_2_/air gas mixture. Cells were routinely subcultured using trypsin solution. CK2 inhibitors were dissolved in DMSO as 1000× stock solutions, diluted in DMEM and added to cells. Control cells were also treated with 0.1% DMSO. TBBz and DMAT were synthesized and kindly provided by Dr. Maria Bretner at the Institute of Biochemistry and Biophysics, Polish Academy of Sciences, Warsaw, Poland.

### Cell proliferation and viability assays

Cell growth was determined by incorporation of [^3^H] thymidine into DNA of proliferating cells and cell viability was monitored by using 3-(4,5-dimethylthiazol-2-yl)-3,5-diphenyltriazolum bromide (MTT) reagent. Exponentially growing cells were harvested, seeded at a density 5 × 10^3 ^cells per well in 96-well plates and grown for 24 h in DMEM containing 10% FBS. Then, cells were supplemented with fresh medium without or with the CK2 inhibitors at the indicated concentrations and 24 h later either 0.1 μCi of [^3^H]thymidine (GE Healthcare) or MTT CellTiter 96 (Promega) were added to each well, as previously described [44]. Four independent experiments were performed and all assays were repeated in octuplicate. Results were expressed as the percentage of control cells (means ± SD).

### Phosphorylation assays

cDNA of hnRNP K protein were subcloned into pET-28(+) expression vector (Novagen), and the plasmid was transformed into *E. coli *BL21 DE3 pLysS cells (Novagen) [20]. Bacterially expressed recombinant proteins were purified by affinity chromatography using Ni-NTA agarose (Qiagen) according to manufacturer's protocol. Nuclear extract (NE) was extracted as described previously [45].

0.1 μg of hnRNP K protein or 6 μg of NE proteins were phosphorylated using CK2 purified from rat liver (Sigma; C3460) or autophosphorylated, respectively, in a final volume 25 μl containing 25 mM Tris, pH = 7.5, 150 mM NaCl, 0.1 mM ATP, 0.1 μCi γ^32^P- ATP, 10 mM MgCl_2 _for 20 min at 30°C, as described previously [20], without or in the presence of the inhibitor. Assays were stopped by boiling with 25 μl 1 × Laemmli loading buffer. Proteins were separated by SDS-PAGE, dried gels were exposed to phosphor screen, scanned using Phosphorimager and densitometrically analyzed.

Sixty μg of NE proteins in immunoprecipitation (IP) buffer [150 mM NaCl, 5 mM EDTA, 1% Triton X-100, 0.5% NP-40, 50 mM Tris-HCl, pH = 7.5, containing the protease (Roche) and phosphatase inhibitors (Sigma)] were incubated with 1 μg of an anti-CDK9 and 1 μg of anti-cyclin T1 antibody (D-7 and H-245, Santa Cruz Biotechnology, respectively) at 4°C for one hour. The complexes were pulled-down by adding Dynabeads^® ^Protein G Magnetic Beads (Invitrogen) (20 μl) and rotating the slurry for 45 min (4°C). Beads were washed three times with 1 ml of IP buffer and two times with 25 mM Tris-HCl, pH = 7.5, and then used for phosphorylation assays.

Beads or 0.1 μg of recombinant fusion full-length human CDK9+CyclinK protein, co-expressed by baculovirus in Sf9 insect cells (Abcam, ab70320) were incubated in 50 μl of phosphorylation buffer at 25°C in an Eppendorf Thermomixer alone or in the presence of 75 mM of peptide substrate (YSPTSPS) for 20 minutes. The assays were terminated by either washing the beads with IP buffer and boiling in 1× Laemmli loading buffer or by boiling the reaction mixture in 2× Laemmli loading buffer and separated by SDS-PAGE. The phosphorylation assays employing YSPTSPS peptide were stopped by adding an equal volume of 2 M HCl followed by acidic hydrolysis of γ^32^P- ATP and separation of ^32^Pi from phosphopeptide according to the protocol described by Ruzzene and Pinna [46].

### Western blotting

Equal amounts of cellular protein were separated by 10% SDS-PAGE, electro-transferred to PVDF membrane and immunostained by standard methods.

### Chromatin immunoprecipitation (ChIP) assay

HeLa cells were grown in plastic 6-well culture plates to 50-60% confluence, then made quiescent by lowering FBS concentration in the medium to 0.5%. Cells were treated 48 hours later with warmed (37°C) DME media supplemented with 15% FBS and containing either DMSO or inhibitors (TBBz - 25 μM, DMAT - 10 μM, DRB - 50 μM) for 5, 15, 30, 60 and 180 min. Chromatin complexes were crosslinked by adding formaldehyde to the culture medium (1.5%, 15 min, RT) at the time points indicated above. Glycine (0.125 M) was then added to plates for 5 min to quench formaldehyde. Cells were collected and chromatin was prepared as described before [47]. Ultrasound treatment was done in Bioruptor (Diagenode, Philadephia, PA) using 30 s on-off cycles for 15 min at high intensity. Chromatin immunoprecipitation assays were performed using the Matrix-ChIP platform as previously described [47]. Briefly, polystyrene 96-well flat-bottom plates washed once with 200 μl PBS/well were incubated overnight at RT with 0.2 mg Protein A in 100 μl PBS/well. After a wash (200 μl PBS/well), the well walls were blocked with 200 μl blocking buffer (30 min, RT). The wells were cleared and are incubated with 0.5 μg RNA polymerase II CTD antibody (Abcam; ab5408 or Santa Cruz; sc-47701 ) diluted in 100 μl of blocking buffer/well (60 min, RT). Chromatin samples (4 μl chromatin/100 μl blocking buffer) were added to wells (100 μl/well) and plates were floated in ultrasonic water bath (60 min, 4°C) to accelerate protein-antibody binding. Wells were washed 3 times with 200 μl IP buffer and once with 200 μl TE buffer. Wells were incubated with 100 μl elution buffer for 15 min at 60°C, followed by 15 min at 95°C. DNA samples were stored at -20°C in the same Matrix-ChIP plates for repeated use.

PCR reaction mixture contained 2.5 μl 2× SYBR Green PCR master mix (SensiMix, Quantace), 2.4 μl DNA template and 0.1 μl primers (200 nM each) in 5 μl final volume in 384-Well Optical Reaction Plate (Applied Biosystems). Amplification (two step, 40 cycles), data acquisition and analysis were done using the 7900HT Real Time PCR system (Applied Biosystems). All PCR reactions were done in triplicates. The primers sequences are available on request. ChIP DNA data are expressed as percent of input DNA, as described before [47].

### Sample preparation and microarray hybridization

HeLa cells were made quiescent by lowering FBS concentration in the medium to 0.5%. 48 h later, the medium was supplemented with 15% FBS and cells were grown with or without CK2 inhibitors. Control cells were treated without or with 0.1% DMSO. At the indicated time points, cells were harvested, and total RNA was extracted using the RNeasy Mini Kit (Qiagen) according to the manufacturer's protocol.

Gene expression analysis was carried out using the Affymetrix U133A 2.0 GeneChip oligo-microarrays containing a total number of 22,277 probe sets, which allows for analyzing the expression level of 18,400 transcripts and variants, including 14,500 well-characterized human genes. Microarray hybridization was performed as described before [48]. Detailed description of array data analysis and results is deposited in Additional file 1. Specific RNA concentrations were also quantified by reverse transcription - real-time PCR [48].

### Statistical analysis

Results are presented as means +/- SD. Significant differences between mean values were assessed by the two-tailed *t*-test for unpaired data using Statistica PL software. Means were considered to be statistically distinct if *p *< 0.05.

### Supporting material

MIAME compliment microarray data are available at http://www.integromics.pl/files/CK2inhibitors/

## Authors' contributions

MM carried out ChIP studies and drafted the manuscript. KH and AD carried out cell tissue culture and phosphorylation studies. AP performed both RT and microarray measurements. TR performed microarray data analyses and participated in the drafting of the manuscript. KB participated in the drafting of the manuscript. JO conceived and designed of the study and drafted the manuscript. All authors read and approved the final manuscript.

## Supplementary Material

Additional file 1 **Microarray data processing and results**. A detailed description of microarray data normalization, filtering and the results. Click here for file
