# Supplementary material for: Halogenated imidazole derivatives block RNA polymerase II elongation along mitogen inducible genes
Source: BMC Mol Biol. 2010 Jan 15;11:4. doi: 10.1186/1471-2199-11-4 (PMC2824761; doi:10.1186/1471-2199-11-4)
Supplement: Additional file 1 — Microarray data processing and results. A detailed description of microarray data normalization, filtering and the results. [file 1471-2199-11-4-S1.PDF]

Halogenated imidazole derivatives block RNA polymerase II elongation along mitogen inducible genes

Michal Mikula, Karolina Hanusek, Agnieszka Paziewska, Artur Dzwonek, Tymon Rubel, Karol Bomsztyk, Jerzy Ostrowski

Suplemental methods

Microarray data processing and analysis

Probe set data (cell intensity files) were generated with Affymetrix Microarray Suite (MAS) 5.0 software [1]. To obtain the gene relative-expression levels, probe set-level data extraction was performed with the GCRMA normalization algorithm implemented in the R package *gcrma* (the Bioconductor project; http://www.bioconductor.org) [2, 3]. The measured expression levels were log transformed (log_2). For data filtration, we selected the probe sets exhibiting signal intensity above the threshold limit, which was established as the 98th percentile of the detectable signals from Y-chromosome-linked probe sets.

For gene-by-gene statistical testing, we used parametric tests, both with equal and unequal variance, to compare differences between groups. Because experimental variability commonly appears among microarray data sets, for multiple testing of the resulting *P*-value significance, the False Discovery Rate (FDR) was employed using the Benjamini-Hochberg procedure. Then, principal component analysis (PCA) and hierarchical clustering were used to evaluate the relationships between samples.

To obtain the gene expression measurements, probe-level data extraction was performed with GC-RMA. The internal consistency of the data sets was also tested using principal component analysis (PCA), normalized unscaled standard error (NUSE) plots and relative log expression (RLE) plots. After initial assessment, none of the arrays were rejected due to sample RNA degradation and poor technical chip quality. For data filtration, we selected the probe sets exhibiting signal intensity above the threshold in at least 2 samples. The threshold was established as the 98th percentile of the expression levels from Y-chromosome-linked probe sets signals detectable in female (HeLa) cells. The total number of remaining probe sets after the filtering step was 10,600. Unsupervised average-linkage hierarchical clustering with a Pearson correlation coefficient based distance metric and PCA were user for graphical summarization and evaluation of the relationships between studied groups of samples after the filtration procedure.

Microarray results

For each time point, the control and DMSO samples were merged in a single group (Figure. 1S). Although a small number of probe sets (from 9 to 17, depending on the time point) were identified as differentially expressed when very permissive criteria were used (p-value of the t-test and fold change thresholds equal to 0.01 and 1.25 respectively), their numbers were in every case smaller than the mean numbers of probe sets obtained after reassigning the samples between groups. So it can be stated that the effect of DMSO is marginal or not detectable due to small

number of samples. In further analyses both untreated and DMSO treated samples were considered together as control groups.

Control groups comparison.

First, we established fetal bovine serum (FBS)-induced gene expression signatures. Of the 10,600 probe sets, ANOVA revealed 925 probe sets that differentially changed at least 2-fold (FDR=0.001) at one of the analyzed time points in combined arrays from control untreated and control DMSO-treated cells. Supplementary Table S1 gives the complete list of probe sets that were differentially expressed after serum treatment.

An evolutionary-driven k-means clustering algorithm with a distance metric derived from Pearson correlation coefficient showed that the 925 probe sets formed 10 distinct clusters of clearly separated expression patterns during FBS treatment (Figure.2S). The selection of the number of clusters was based on visual inspection of the dendrogram resulting from an average-linkage hierarchical clustering procedure executed prior to the main cluster analysis. Seventy one percent of the differentially expressed probe sets showed a peak increase in expression at 1 or 6 hrs after serum treatment.

Inhibitor vs. control groups comparison

As shown in Supplementary Figure. 3S (A) while all arrays from the one-hour experimental time point clustered together, arrays from cells treated with CK2 inhibitors for 6 and 24 hr were notably distinct from the control cells. Pair-wise comparisons between the inhibitor-treated and untreated cells were performed. At each time point, differential probe sets were identified using a t-test for significance along with the requirement of at least a 3-fold change in expression. Due to the small number of samples the t-test was used as a filter for removing those probe sets with large fold changes caused by extreme values in single samples. The p-value threshold was equal to 0.01 and no false discovery rate control procedure was used in this analysis.

1. GeneChip® Expression Analysis – Data Analysis Fundamentals [http://www.affymetrix.com/support/downloads/manuals/data_analysis_fundamentals_manual.pdf]
2. Wu ZJ, Irizarry RA, Gentleman R, Martinez-Murillo F, Spencer F: **A model-based background adjustment for oligonucleotide expression arrays**. *J Am Stat Assoc* 2004, 99(468):909-917.
3. Gentleman RC, Carey VJ, Bates DM, Bolstad B, Dettling M, Dudoit S, Ellis B, Gautier L, Ge Y, Gentry J *et al*: **Bioconductor: open software development for computational biology and bioinformatics**. *Genome biology* 2004, 5(10):R80.

Supplemental figures

Figure. S1 Visualization of sample distribution obtained by PCA (A) and result (B) of hierarchical clusterization (based on 10600 probe sets left after filtration).

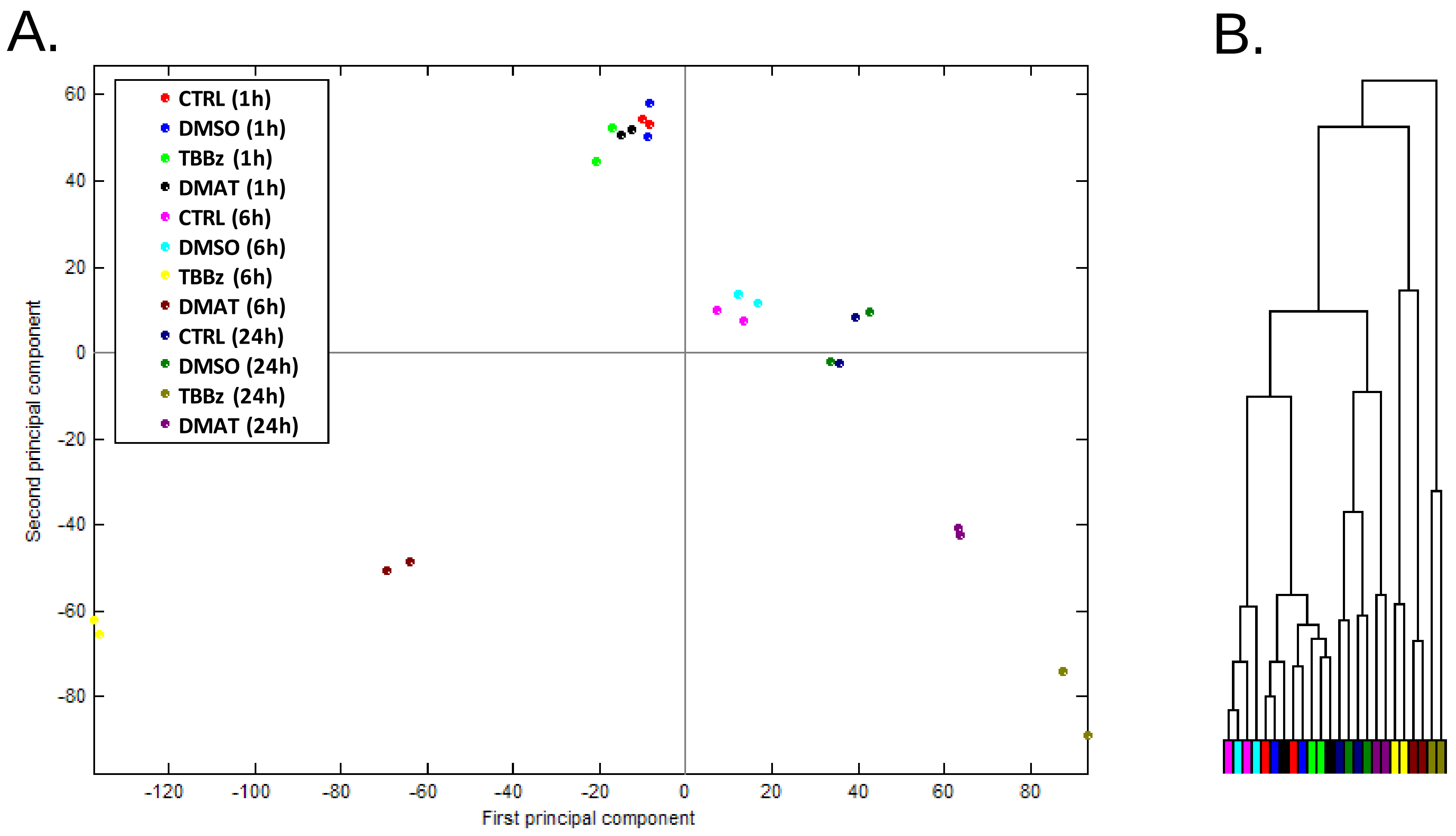

Figure. S2 Hierarchical clustering forms 10 distinct clusters of clearly separated expression patterns during cell treatment with FBS.

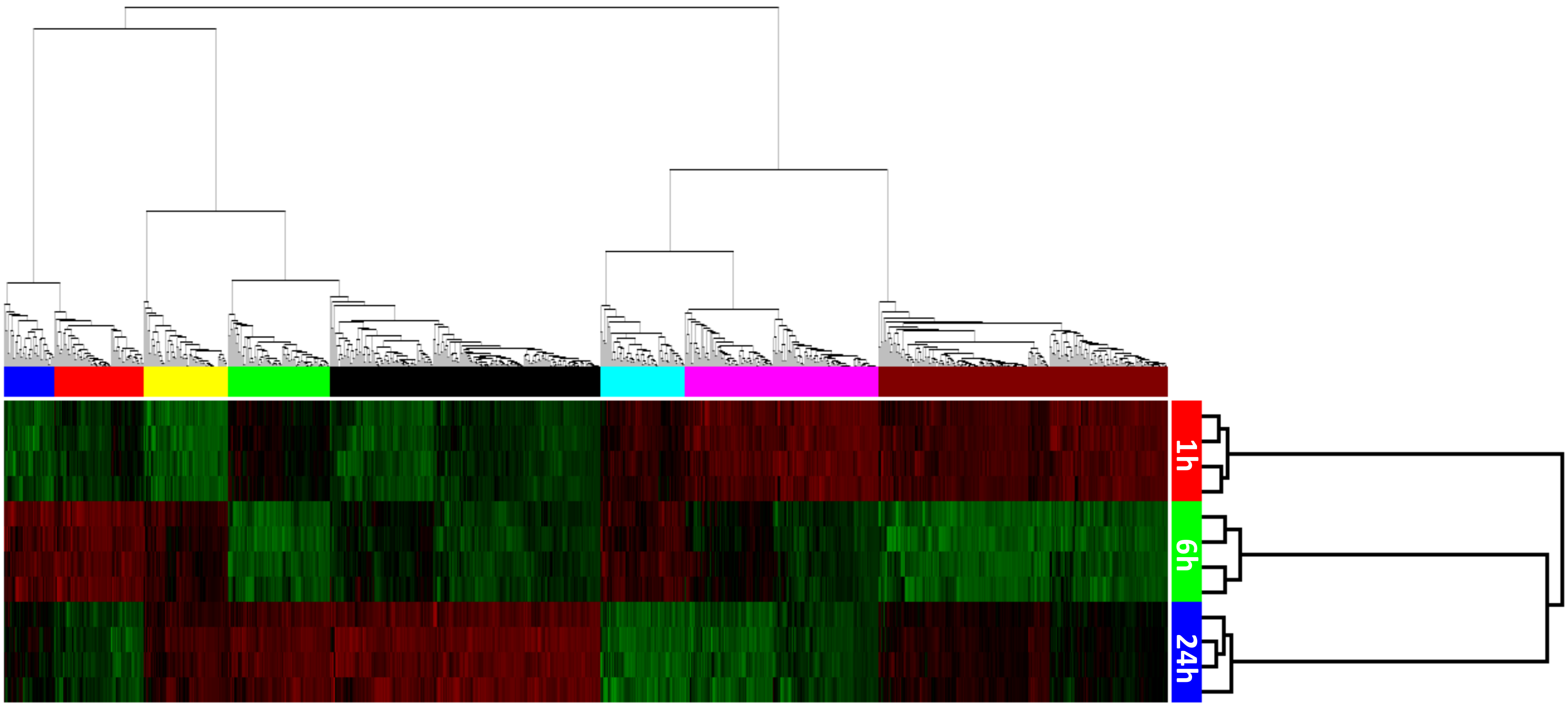

Figure. S3 Principal component analysis (A) and hierarchical clustering (B) computed from 10600 probe sets which remained after data filtration.

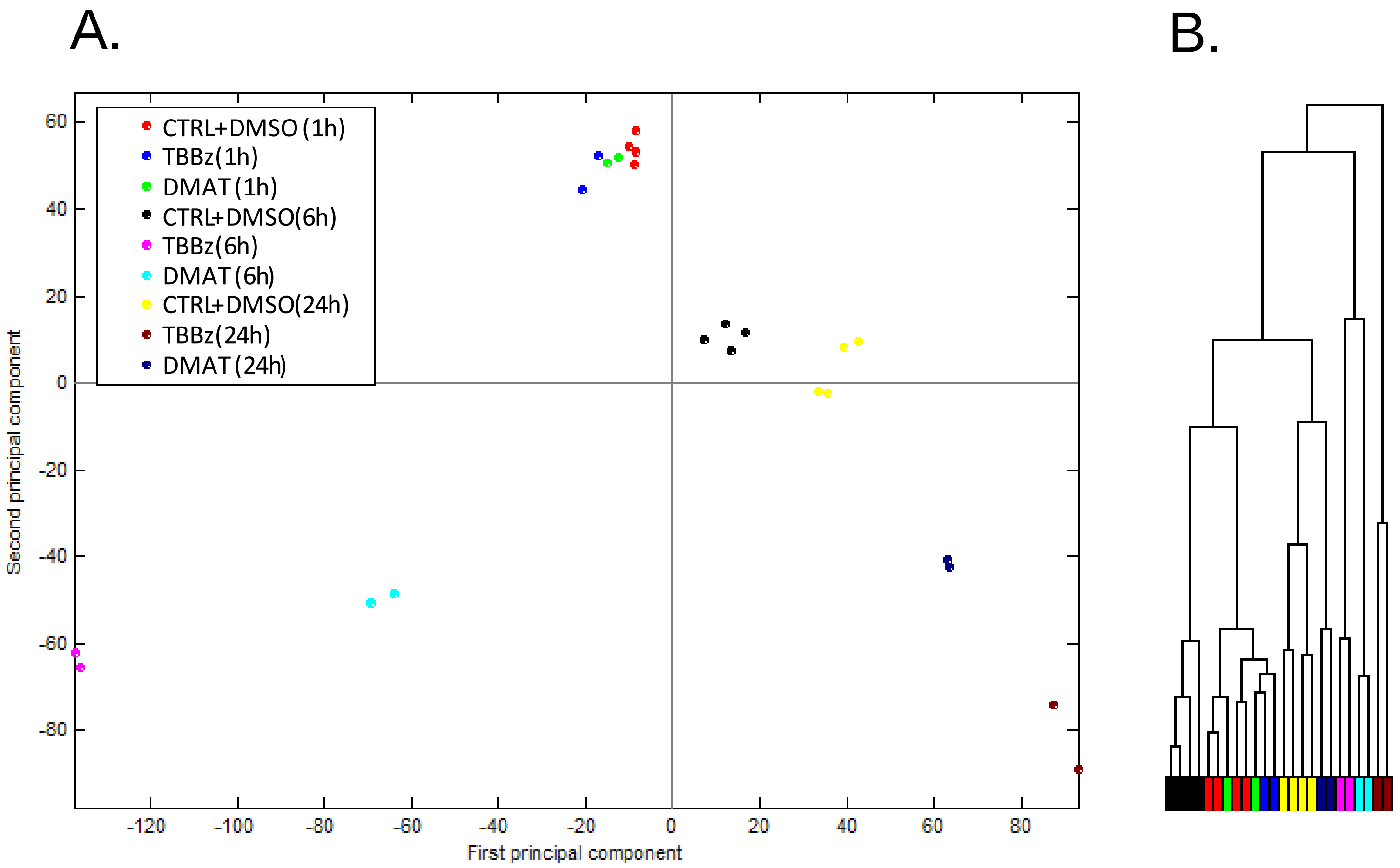

Table S1. List of probe sets differentially expressed after serum treatment.

Probe	GenBank	Symbol	Description	1h	6h	24h	ANOVA	FDR	Chromosome
202768_at	NM_006732	FOSB	FBJ murine osteosarcoma viral oncogene homolog B	246.506	4.512	4.496	6.66E-16	7.06E-12	19
205249_at	NM_000399	EGR2	early growth response 2 (Krox-20 homolog, Drosophila)	482.66	5.611	5.545	2.44E-14	1.29E-10	10
201236_s_at	BG339064	BTG2	BTG family, member 2	1123.822	22.403	20.706	2.36E-13	7.34E-10	1
202672_s_at	NM_001674	ATF3	activating transcription factor 3	5131.49	399.812	261.965	3.28E-13	7.34E-10	1
202068_s_at	NM_000527	LDLR	low density lipoprotein receptor (familial hypercholesterolemia)	5270.595	351.022	664.372	3.46E-13	7.34E-10	19
202540_s_at	NM_000859	HMGCR	3-hydroxy-3-methylglutaryl-Coenzyme A reductase	3671.037	797.206	1198.844	4.52E-13	7.99E-10	5
201627_s_at	BE300521	INSIG1	insulin induced gene 1	3573.833	195.716	257.496	8.30E-13	1.11E-09	7
204677_at	NM_001795	CDH5	cadherin 5, type 2, VE-cadherin (vascular epithelium)	5.098	33.819	5.045	8.86E-13	1.11E-09	16
217997_at	NM_007350	PHLDA1	pleckstrin homology-like domain, family A, member 1	111.265	110.108	5.501	9.84E-13	1.11E-09	12
209101_at	M92934	CTGF	connective tissue growth factor	12969.513	1582.649	76.661	1.05E-12	1.11E-09	6
201693_s_at	NM_001964	EGR1	early growth response 1	400.344	4.394	4.17	1.79E-12	1.73E-09	5
201466_s_at	BC002646	JUN	jun oncogene	490.22	55.506	6.589	3.42E-12	3.02E-09	1
209230_s_at	AF135266	NUPR1	nuclear protein 1	261.214	191.82	705.571	4.11E-12	3.11E-09	16
209146_at	U93162	SC4MOL	sterol-C4-methyl oxidase-like	6557.44	750.556	1171.391	4.11E-12	3.11E-09	4
221541_at	AL136861	CRISPLD2	cysteine-rich secretory protein LCCL domain containing 2	5.288	128.586	5.332	6.03E-12	4.15E-09	16
209608_s_at	BC000408	ACAT2	acetyl-Coenzyme A acetyltransferase 2	5519.74	4287.003	1417.996	6.35E-12	4.15E-09	6
216598_s_at	S69738	CCL2	chemokine (C-C motif) ligand 2	508.216	703.039	16.202	6.65E-12	4.15E-09	17
202340_x_at	NM_002135	NR4A1	nuclear receptor subfamily 4, group A, member 1	2098.674	34.706	21.649	8.02E-12	4.72E-09	12
202643_s_at	AI738896	TNFAIP3	tumor necrosis factor, alpha-induced protein 3	832.095	241.595	16.657	8.98E-12	5.01E-09	6
217996_at	NM_007350	PHLDA1	pleckstrin homology-like domain, family A, member 1	564.819	613.475	24.374	9.67E-12	5.12E-09	12
202644_s_at	NM_006290	TNFAIP3	tumor necrosis factor, alpha-induced protein 3	1544.809	444.286	63.489	1.10E-11	5.54E-09	6

201531_at	NM_003407	ZFP36	zinc finger protein 36, C3H type, homolog (mouse)	308.249	34.018	15.666	1.36E-11	6.43E-09	19
204621_s_at	AI935096	NR4A2	nuclear receptor subfamily 4, group A, member 2	4753.221	75.605	127.375	1.40E-11	6.43E-09	2
221843_s_at	AA195017	KIAA1609	KIAA1609	5.522	24.34	5.547	2.18E-11	9.63E-09	16
215111_s_at	AK027071	TSC22D1	TSC22 domain family, member 1	14264.083	6719.975	7917.482	2.86E-11	1.21E-08	13
201289_at	NM_001554	CYR61	cysteine-rich, angiogenic inducer, 61	20894.044	12263.7	1805.527	4.40E-11	1.73E-08	1
208647_at	AA872727	FDFT1	farnesyl-diphosphate farnesyltransferase 1	12194.617	8040.716	3962.925	4.78E-11	1.81E-08	8
208881_x_at	BC005247	IDI1	isopentenyl-diphosphate delta isomerase 1	10223.949	3285.543	1501.789	5.16E-11	1.83E-08	10
204602_at	NM_012242	DKK1	dickkopf homolog 1 (Xenopus laevis)	13.298	1040.782	22.805	5.29E-11	1.83E-08	10
201625_s_at	BE300521	INSIG1	insulin induced gene 1	1171.447	293.641	89.509	5.38E-11	1.83E-08	7
206969_at	NM_021013	KRT34	keratin 34	5.807	97.788	5.121	5.51E-11	1.83E-08	17
205476_at	NM_004591	CCL20	chemokine (C-C motif) ligand 20	48.855	391.576	4.415	5.73E-11	1.84E-08	2
204615_x_at	NM_004508	IDI1	isopentenyl-diphosphate delta isomerase 1	9455.832	3168.812	1509.468	6.49E-11	2.02E-08	10
205047_s_at	NM_001673	ASNS	asparagine synthetase	843.162	886.085	2791.424	7.36E-11	2.23E-08	7
36711_at	AL021977	MAFF	v-maf musculoaponeurotic fibrosarcoma oncogene homolog F (avian)	3147.565	2359.934	198.365	7.59E-11	2.24E-08	22
65517_at	AA910946	AP1M2	adaptor-related protein complex 1, mu 2 subunit	1146.502	874.982	356.073	8.04E-11	2.29E-08	19
210764_s_at	AF003114	CYR61	cysteine-rich, angiogenic inducer, 61	12650.448	4738.555	512.915	8.22E-11	2.29E-08	1
204622_x_at	NM_006186	NR4A2	nuclear receptor subfamily 4, group A, member 2	3483.442	33.197	73.001	8.53E-11	2.32E-08	2
212242_at	AL565074	TUBA4A	tubulin, alpha 4a	1585.361	3991.181	4551.825	1.12E-10	2.96E-08	2
204533_at	NM_001565	CXCL10	chemokine (C-X-C motif) ligand 10	130.384	42.367	6.171	1.16E-10	2.99E-08	4
200831_s_at	AA678241	SCD	stearoyl-CoA desaturase (delta-9-desaturase)	8215.274	6301.475	1697.707	1.19E-10	2.99E-08	10
205193_at	NM_012323	MAFF	v-maf musculoaponeurotic fibrosarcoma oncogene homolog F (avian)	230.55	169.256	13.784	1.21E-10	2.99E-08	22
209457_at	U16996	DUSP5	dual specificity phosphatase 5	3439.278	128.317	8.337	1.39E-10	3.35E-08	10

212665_at	AL556438	TIPARP	TCDD-inducible poly(ADP-ribose) polymerase	1536.961	689.364	326.698	1.46E-10	3.43E-08	3
201170_s_at	NM_003670	BHLHB2	basic helix-loop-helix domain containing, class B, 2	6710.485	2013.682	1959.965	1.96E-10	4.52E-08	3
207978_s_at	NM_006981	NR4A3	nuclear receptor subfamily 4, group A, member 3	1395.468	43.202	41.95	2.01E-10	4.52E-08	9
209959_at	U12767	NR4A3	nuclear receptor subfamily 4, group A, member 3	3330.83	178.365	186.712	2.11E-10	4.67E-08	9
201109_s_at	AV726673	THBS1	thrombospondin 1	703.136	3795.992	201.801	2.40E-10	5.18E-08	15
218205_s_at	NM_017572	MKNK2	MAP kinase interacting serine/threonine kinase 2	2573.355	1052.986	2898.005	2.67E-10	5.59E-08	19
210950_s_at	BC003573	FDFT1	farnesyl-diphosphate farnesyltransferase 1	11461.321	7596.06	3029.694	2.69E-10	5.59E-08	8
201631_s_at	NM_003897	IER3	immediate early response 3	5868.384	2892.03	698.229	3.13E-10	6.39E-08	6
210095_s_at	M31159	IGFBP3	insulin-like growth factor binding protein 3	72.025	465.91	217.788	3.41E-10	6.82E-08	7
202613_at	NM_001905	CTPS	CTP synthase	948.095	3824.265	1166.954	3.55E-10	6.86E-08	1
204790_at	NM_005904	SMAD7	SMAD family member 7	15.734	55.998	56.298	3.56E-10	6.86E-08	18
212412_at	AV715767	PDLIM5	PDZ and LIM domain 5	1658.004	4444.207	1509.506	3.67E-10	6.94E-08	4
204420_at	BG251266	FOSL1	FOS-like antigen 1	282.755	444.416	13.562	3.84E-10	7.13E-08	11
212190_at	AL541302	SERPINE2	serpin peptidase inhibitor, clade E (nexin, plasminogen activator inhibitor type 1), member 2	425.455	1736.79	2106.356	4.11E-10	7.51E-08	2
220081_x_at	NM_016371	HSD17B7	hydroxysteroid (17-beta) dehydrogenase 7	2120.571	1132.874	411.085	5.13E-10	9.21E-08	1
202081_at	NM_004907	IER2	immediate early response 2	9990.595	2311.952	1601.623	5.32E-10	9.40E-08	19
209774_x_at	M57731	CXCL2	chemokine (C-X-C motif) ligand 2	485.544	197.031	16.015	5.56E-10	9.66E-08	4
211143_x_at	D49728	NR4A1	nuclear receptor subfamily 4, group A, member 1	139.496	8.514	8.096	6.63E-10	1.13E-07	12
202539_s_at	NM_000859	HMGCR	3-hydroxy-3-methylglutaryl-Coenzyme A reductase	6304.774	1555.237	2054.83	8.41E-10	1.37E-07	5
208370_s_at	NM_004414	RCAN1	regulator of calcineurin 1	2153.181	1214.451	979.438	1.06E-09	1.68E-07	21
213906_at	AW592266	MYBL1	v-myb myeloblastosis viral oncogene homolog (avian)-like 1	827.696	3645.125	749.234	1.20E-09	1.84E-07	8

218856_at	NM_016629	TNFRSF21	tumor necrosis factor receptor superfamily, member 21	100.186	412.978	99.984	1.20E-09	1.84E-07	6
213280_at	AK000478	GARNL4	GTPase activating Rap/RanGAP domain-like 4	780.161	2197.953	773.82	1.21E-09	1.84E-07	17
201790_s_at	AW150953	DHCR7	7-dehydrocholesterol reductase	844.773	531.836	175.417	1.25E-09	1.86E-07	11
205207_at	NM_000600	IL6	interleukin 6 (interferon, beta 2)	1510.341	789.858	39.615	1.29E-09	1.90E-07	7
201108_s_at	NM_003246	THBS1	thrombospondin 1	263.779	2176.186	95.085	1.41E-09	2.05E-07	15
202887_s_at	NM_019058	DDIT4	DNA-damage-inducible transcript 4	2274.664	2215.901	8705.796	1.46E-09	2.06E-07	10
204440_at	NM_004233	CD83	CD83 molecule	175.231	269.651	57.752	1.49E-09	2.07E-07	6
206683_at	NM_003447	ZNF165	zinc finger protein 165	196.398	83.4	61.652	1.52E-09	2.07E-07	6
209218_at	AF098865	SQLE	squalene epoxidase	5457.259	2309.996	1395.766	1.52E-09	2.07E-07	8
203108_at	NM_003979	GPRC5A	G protein-coupled receptor, family C, group 5, member A	475.369	2352.616	648.029	1.66E-09	2.22E-07	12
209939_x_at	AF005775	CFLAR	CASP8 and FADD-like apoptosis regulator	367.78	724.044	297.345	1.68E-09	2.22E-07	2
209822_s_at	L22431	VLDLR	very low density lipoprotein receptor	118.07	137.957	446.634	1.71E-09	2.24E-07	9
201041_s_at	NM_004417	DUSP1	dual specificity phosphatase 1	15476.52	5404.671	2002.274	1.77E-09	2.26E-07	5
201464_x_at	BC002646	JUN	jun oncogene	1573.115	305.922	59.147	1.81E-09	2.26E-07	1
217678_at	AA488687	SLC7A11	solute carrier family 7, (cationic amino acid transporter, y+ system) member 11	95.201	94.752	758.415	1.82E-09	2.26E-07	4
217188_s_at	AC007182	C14orf1	chromosome 14 open reading frame 1	289.864	214.427	90.026	1.82E-09	2.26E-07	14
201275_at	NM_002004	FDPS	farnesyl diphosphate synthase (farnesyl pyrophosphate synthetase, dimethylallyltranstransferase, geranyltranstransferase)	14214.403	12256.44	5253.134	1.85E-09	2.26E-07	1
44783_s_at	R61374	HEY1	hairy/enhancer-of-split related with YRPW motif 1	373.32	239.935	817.538	1.86E-09	2.26E-07	8
216248_s_at	S77154	NR4A2	nuclear receptor subfamily 4, group A, member 2	4436.807	52.331	106.877	1.99E-09	2.39E-07	2
203627_at	AI830698	IGF1R	insulin-like growth factor 1 receptor	1487.228	4116.037	2295.4	2.04E-09	2.43E-07	15

204056_s_at	NM_000431	MVK	mevalonate kinase	33.865	9.899	10.299	2.43E-09	2.77E-07	12
201506_at	NM_000358	TGFBI	transforming growth factor, beta-induced, 68kDa	5.825	364.997	21.668	2.50E-09	2.81E-07	5
222088_s_at	AA778684	SLC2A3	solute carrier family 2 (facilitated glucose transporter), member 3	893.844	154.734	468.347	2.53E-09	2.82E-07	12
202067_s_at	AI861942	LDLR	low density lipoprotein receptor (familial hypercholesterolemia)	582.792	31.824	69.407	2.68E-09	2.96E-07	19
201626_at	BG292233	INSIG1	insulin induced gene 1	5436.933	305.612	526.499	2.81E-09	3.07E-07	7
204027_s_at	NM_005371	METTL1	methyltransferase like 1	114.003	282.083	125.977	2.97E-09	3.21E-07	12
204421_s_at	M27968	FGF2	fibroblast growth factor 2 (basic)	249.289	1351.774	185.277	3.02E-09	3.23E-07	4
202562_s_at	AL136658	C14orf1	chromosome 14 open reading frame 1	1016.016	749.255	315.846	3.05E-09	3.23E-07	14
218706_s_at	NM_023927	GRAMD3	GRAM domain containing 3	762.909	2010.702	492.444	3.18E-09	3.34E-07	5
209013_x_at	AF091395	TRIO	triple functional domain (PTPRF interacting)	107.495	325.684	140.332	3.25E-09	3.38E-07	5
208581_x_at	NM_005952	MT1X	metallothionein 1X	2372.669	17161.04	11707.49	3.29E-09	3.38E-07	16
215195_at	AF035594	PRKCA	protein kinase C, alpha	29.248	124.504	86.621	3.70E-09	3.74E-07	17
217028_at	AJ224869	CXCR4	chemokine (C-X-C motif) receptor 4	4799.058	1839.217	2714.816	3.78E-09	3.78E-07	2
202743_at	BE622627	PIK3R3	phosphoinositide-3-kinase, regulatory subunit 3 (gamma)	852.383	227.18	481.596	3.87E-09	3.84E-07	1
205042_at	NM_005476	GNE	glucosamine (UDP-N-acetyl)-2-epimerase/N-acetylmannosamine kinase	979.123	409.037	368.268	4.38E-09	4.30E-07	9
202245_at	AW084510	LSS	lanosterol synthase (2,3-oxidosqualene-lanosterol cyclase)	872.66	525.89	191.142	4.50E-09	4.38E-07	21
212971_at	AI769685	CARS	cysteinyl-tRNA synthetase	1171.591	1247.797	2859.947	4.61E-09	4.40E-07	11
209967_s_at	D14826	CREM	cAMP responsive element modulator	999.935	868.619	444.252	4.61E-09	4.40E-07	10
201278_at	N21202	DAB2	disabled homolog 2, mitogen-responsive phosphoprotein (Drosophila)	989.474	526.14	481.082	4.69E-09	4.43E-07	5
212724_at	BG054844	RND3	Rho family GTPase 3	1675.065	2044.644	802.925	4.77E-09	4.47E-07	2

218807_at	NM_006113	VAV3	vav 3 guanine nucleotide exchange factor	615.432	63.044	497.457	4.84E-09	4.50E-07		1
201791_s_at	NM_001360	DHCR7	7-dehydrocholesterol reductase	628.444	361.725	127.643	4.98E-09	4.56E-07		11
209189_at	BC004490	FOS	v-fos FBJ murine osteosarcoma viral oncogene homolog	885.136	40.5	9.802	5.02E-09	4.56E-07		14
203633_at	BF001714	CPT1A	carnitine palmitoyltransferase 1A (liver)	289.438	282.537	635.144	5.13E-09	4.61E-07		11
218995_s_at	J05008	EDN1	endothelin 1	204.439	48.804	13.389	5.61E-09	4.96E-07		6
203499_at	NM_004431	EPHA2	EPH receptor A2	312	559.031	130.97	5.81E-09	5.09E-07		1
218261_at	NM_005498	AP1M2	adaptor-related protein complex 1, mu 2 subunit	1269.118	1068.759	434.644	6.53E-09	5.67E-07		19
210163_at	AF030514	CXCL11	chemokine (C-X-C motif) ligand 11	62.122	35.841	8.581	6.91E-09	5.95E-07		4
213562_s_at	BF979497	SQLE	squalene epoxidase	889.222	358.027	198.619	7.02E-09	6.00E-07		8
213338_at	BF062629	TMEM158	transmembrane protein 158	441.595	1564.586	509.19	7.20E-09	6.10E-07		3
203233_at	NM_000418	IL4R	interleukin 4 receptor	24.295	70.388	22.26	8.09E-09	6.80E-07		16
208706_s_at	AK026933	EIF5	eukaryotic translation initiation factor 5	2599.975	5610.533	4428.331	8.17E-09	6.82E-07		14
207559_s_at	NM_005096	ZMYM3	zinc finger, MYM-type 3	198.669	88.669	263.468	8.53E-09	7.06E-07	X	
203002_at	NM_016201	AMOTL2	angiomotin like 2	1144.323	2007.582	664.576	8.63E-09	7.09E-07		3
218966_at	NM_018728	MYO5C	myosin VC	990.675	747.474	482.781	9.38E-09	7.51E-07		15
212845_at	AB028976	SAMD4A	sterile alpha motif domain containing 4A	154.164	723.223	115.828	9.42E-09	7.51E-07		14
219863_at	NM_016323	HERC5	hect domain and RLD 5	2463.055	1251.069	958.936	9.64E-09	7.62E-07		4
219071_x_at	NM_016458	C8orf30A	chromosome 8 open reading frame 30A	96.748	141.321	276.227	9.88E-09	7.70E-07		8
201044_x_at	AA530892	DUSP1	dual specificity phosphatase 1	1526.855	464.678	136.117	9.95E-09	7.70E-07		5
203123_s_at	AU154469	SLC11A2	solute carrier family 11 (proton-coupled divalent metal ion transporters), member 2	1301.03	613.817	864.87	1.01E-08	7.71E-07		12
205398_s_at	NM_005902	SMAD3	SMAD family member 3	119.819	250.814	86.783	1.01E-08	7.71E-07		15
222162_s_at	AK023795	ADAMTS1	ADAM metallopeptidase with thrombospondin type 1 motif, 1	789.419	84.493	58.068	1.06E-08	8.04E-07		21

201207_at	NM_021137	TNFAIP1	tumor necrosis factor, alpha-induced protein 1 (endothelial)	847.882	1471.792	508.032	1.10E-08	8.26E-07	17
216804_s_at	AK027217	PDLIM5	PDZ and LIM domain 5	169.507	799.147	229.032	1.19E-08	8.74E-07	4
220892_s_at	NM_021154	PSAT1	phosphoserine aminotransferase 1	1096.572	1096.031	3097.615	1.19E-08	8.74E-07	9
219961_s_at	NM_018474	C20orf19	chromosome 20 open reading frame 19	191.735	34.071	111.352	1.20E-08	8.74E-07	20
202499_s_at	NM_006931	SLC2A3	solute carrier family 2 (facilitated glucose transporter), member 3	1304.254	221.814	707.227	1.20E-08	8.74E-07	12
203340_s_at	AI887457	SLC25A12	solute carrier family 25 (mitochondrial carrier, Aralar), member 12	115.343	62.223	162.32	1.24E-08	8.91E-07	2
201475_x_at	NM_004990	MARS	methionyl-tRNA synthetase	837.812	984.389	2129.683	1.38E-08	9.85E-07	12
206173_x_at	NM_002041	GABPB2	GA binding protein transcription factor, beta subunit 2	169.434	547.77	268.81	1.40E-08	9.87E-07	15
203973_s_at	M83667	CEBPD	CCAAT/enhancer binding protein (C/EBP), delta	1586.461	1017.022	518.216	1.44E-08	1.01E-06	8
214433_s_at	NM_003944	SELENBP1	selenium binding protein 1	588.115	565.983	253.648	1.46E-08	1.01E-06	1
219474_at	NM_024616	C3orf52	chromosome 3 open reading frame 52	149.708	364.531	134.415	1.52E-08	1.05E-06	3
202023_at	NM_004428	EFNA1	ephrin-A1	1702.839	2845.37	888.155	1.54E-08	1.06E-06	1
210162_s_at	U08015	NFATC1	nuclear factor of activated T-cells, cytoplasmic, calcineurin-dependent 1	15.345	53.135	15.402	1.57E-08	1.07E-06	18
204981_at	NM_002555	SLC22A18	solute carrier family 22, member 18	176.55	140.386	73.188	1.71E-08	1.15E-06	11
200832_s_at	AB032261	SCD	stearoyl-CoA desaturase (delta-9-desaturase)	15966.795	11475.47	4869.38	1.92E-08	1.27E-06	10
202959_at	AI433712	MUT	methylmalonyl Coenzyme A mutase	411.222	285.168	141.979	2.01E-08	1.30E-06	6
219940_s_at	NM_018386	PCID2	PCI domain containing 2	1772.332	4286.999	2367.957	2.02E-08	1.30E-06	13
1405_i_at	M21121	CCL5	chemokine (C-C motif) ligand 5	734.898	493.386	130.352	2.03E-08	1.30E-06	17
202949_s_at	NM_001450	FHL2	four and a half LIM domains 2	4063.183	9854.511	3719.456	2.13E-08	1.36E-06	2
212185_x_at	NM_005953	MT2A	metallothionein 2A	7141.221	30569.47	23532.71	2.19E-08	1.38E-06	16
209034_at	AF279899	PNRC1	proline-rich nuclear receptor	791.492	362.67	497.876	2.19E-08	1.38E-06	6

			coactivator 1						
213577_at	AA639705	SQLE	squalene epoxidase	627.358	168.324	107.701	2.24E-08	1.40E-06	8
222108_at	AC004010	AMIGO2	adhesion molecule with Ig-like domain 2	127.752	982.946	186.205	2.30E-08	1.43E-06	12
208693_s_at	D30658	GARS	glycyl-tRNA synthetase	2696.306	2743.664	6766.9	2.36E-08	1.45E-06	7
210845_s_at	U08839	PLAUR	plasminogen activator, urokinase receptor	455.865	270.406	214.007	2.51E-08	1.51E-06	19
204715_at	NM_015368	PANX1	pannexin 1	319.124	504.799	211.753	2.59E-08	1.55E-06	11
200779_at	NM_001675	ATF4	activating transcription factor 4 (tax-responsive enhancer element B67)	3854.684	3732.371	10250.27	2.60E-08	1.55E-06	22
211980_at	AI922605	COL4A1	collagen, type IV, alpha 1	335.477	996.358	827.906	2.61E-08	1.55E-06	13
209102_s_at	AF019214	HBP1	HMG-box transcription factor 1	705.71	225.076	280.506	2.74E-08	1.61E-06	7
201694_s_at	NM_001964	EGR1	early growth response 1	2663.312	105.807	8.887	2.74E-08	1.61E-06	5
204821_at	NM_006994	BTN3A3	butyrophilin, subfamily 3, member A3	200.161	66.952	95.397	2.85E-08	1.65E-06	6
201502_s_at	NM_020529	NFKBIA	nuclear factor of kappa light polypeptide gene enhancer in B-cells inhibitor, alpha	3888.256	2478.289	842.057	2.92E-08	1.68E-06	14
209304_x_at	AF087853	GADD45B	growth arrest and DNA-damage-inducible, beta	468.158	267.212	99.208	2.99E-08	1.69E-06	19
208966_x_at	AF208043	IFI16	interferon, gamma-inducible protein 16	902.675	420.6	885.905	3.02E-08	1.69E-06	1
222148_s_at	BF688108	RHOT1	ras homolog gene family, member T1	770.821	337.982	737.333	3.04E-08	1.70E-06	17
212197_x_at	AB020671	M-RIP	myosin phosphatase-Rho interacting protein	450.719	785.63	991.532	3.16E-08	1.75E-06	17
50965_at	AI690165	RAB26	RAB26, member RAS oncogene family	60.305	31.447	17.781	3.19E-08	1.76E-06	16
212048_s_at	AW245400	YARS	tyrosyl-tRNA synthetase	1135.745	1148.482	2300.378	3.26E-08	1.79E-06	1
211538_s_at	U56725	HSPA2	heat shock 70kDa protein 2	469.489	718.765	212.107	3.40E-08	1.86E-06	14
208964_s_at	AL512760	FADS1	fatty acid desaturase 1	3855.119	2845.448	1453.673	3.56E-08	1.93E-06	11
203243_s_at	NM_006457	PDLIM5	PDZ and LIM domain 5	590.927	1880.295	594.139	3.61E-08	1.95E-06	4
204655_at	NM_002985	CCL5	chemokine (C-C motif) ligand 5	663.785	465.552	121.441	3.66E-08	1.95E-06	17

203725_at	NM_001924	GADD45A	growth arrest and DNA-damage-inducible, alpha	624.548	1215.961	389.157	3.92E-08	2.05E-06	1
217783_s_at	NM_016061	YPEL5	yippee-like 5 (Drosophila)	859.145	611.868	297.432	3.92E-08	2.05E-06	2
217875_s_at	NM_020182	TMEPAI	transmembrane, prostate androgen induced RNA	15.5	62.77	22.959	4.00E-08	2.07E-06	20
220780_at	NM_015715	PLA2G3	phospholipase A2, group III	306.623	80.496	96.498	4.10E-08	2.10E-06	22
212444_at	AA156240			1063.437	3174.167	863.616	4.23E-08	2.13E-06	
37028_at	U83981	PPP1R15A	protein phosphatase 1, regulatory (inhibitor) subunit 15A	131.036	58.975	32.72	4.34E-08	2.17E-06	19
201110_s_at	NM_003246	THBS1	thrombospondin 1	2962.309	9626.223	855.84	4.77E-08	2.36E-06	15
202149_at	AL136139	NEDD9	neural precursor cell expressed, developmentally down-regulated 9	670.085	1107.468	212.69	4.82E-08	2.37E-06	6
218145_at	NM_021158	TRIB3	tribbles homolog 3 (Drosophila)	928.572	459.291	997.752	5.09E-08	2.49E-06	20
202402_s_at	NM_001751	CARS	cysteinyl-tRNA synthetase	181.01	206.433	592.902	5.15E-08	2.50E-06	11
205100_at	NM_005110	GFPT2	glutamine-fructose-6-phosphate transaminase 2	149.557	289.476	90.166	5.35E-08	2.58E-06	5
215499_at	AA780381	MAP2K3	mitogen-activated protein kinase kinase 3	218.59	572.131	266.452	5.44E-08	2.61E-06	17
219429_at	NM_024306	FA2H	fatty acid 2-hydroxylase	22.671	9.088	15.089	5.48E-08	2.61E-06	16
212143_s_at	BF340228	IGFBP3	insulin-like growth factor binding protein 3	7.785	66.987	25.754	5.49E-08	2.61E-06	7
218368_s_at	NM_016639	TNFRSF12A	tumor necrosis factor receptor superfamily, member 12A	350.423	1859.899	559.781	5.59E-08	2.64E-06	16
203751_x_at	NM_005354	JUND	jun D proto-oncogene	127.312	37.565	40.934	5.74E-08	2.70E-06	19
208891_at	BC003143	DUSP6	dual specificity phosphatase 6	135.936	75.027	17.568	5.88E-08	2.76E-06	12
204897_at	AA897516	PTGER4	prostaglandin E receptor 4 (subtype EP4)	1987.472	656.89	369.897	5.91E-08	2.76E-06	5
212828_at	AA191573	SYNJ2	synaptojanin 2	47.297	127.641	47.714	6.03E-08	2.81E-06	6
218611_at	NM_016545	IER5	immediate early response 5	3086.768	2336.522	1280.821	6.15E-08	2.83E-06	1
220987_s_at	NM_030952	NUAK2	NUAK family, SNF1-like kinase, 2	1630.673	336.244	88.792	6.18E-08	2.84E-06	1
200758_s_at	NM_003204	NFE2L1	nuclear factor (erythroid-derived 2)-like 1	632.86	472.945	1079.148	6.22E-08	2.84E-06	17
201490_s_at	NM_005729	PPIF	peptidylprolyl isomerase F (cyclophilin F)	1569.736	3423.366	1989.336	6.54E-08	2.96E-06	10

210004_at	AF035776	OLR1	oxidized low density lipoprotein (lectin-like) receptor 1	765.813	2110.375	573.104	6.74E-08	3.00E-06	12
212276_at	D80010	LPIN1	lipin 1	263.537	99.396	115.06	6.96E-08	3.08E-06	2
213260_at	AU145890			547.459	440.575	251.241	7.38E-08	3.21E-06	
205330_at	NM_002430	MN1	meningioma (disrupted in balanced translocation) 1	368.839	271.539	600.265	7.39E-08	3.21E-06	22
200931_s_at	NM_014000	VCL	vinculin	2737.744	4832.996	2076.836	7.39E-08	3.21E-06	10
213629_x_at	BF246115	MT1F	metallothionein 1F	61.742	410.287	278.21	7.49E-08	3.23E-06	16
202071_at	NM_002999	SDC4	syndecan 4	3241.608	5034.379	1194.542	8.04E-08	3.44E-06	20
205205_at	NM_006509	RELB	v-rel reticuloendotheliosis viral oncogene homolog B, nuclear factor of kappa light polypeptide gene enhancer in B-cells 3 (avian)	6.341	23.933	4.518	9.15E-08	3.84E-06	19
217165_x_at	M10943	MT1F	metallothionein 1F	824.47	5710.647	3597.864	9.23E-08	3.84E-06	16
202076_at	NM_001166	BIRC2	baculoviral IAP repeat-containing 2	1547.049	3437.776	1328.604	9.26E-08	3.84E-06	11
209824_s_at	AB000812	ARNTL	aryl hydrocarbon receptor nuclear translocator-like	65.772	186.351	68.236	9.28E-08	3.84E-06	11
221750_at	BG035985	HMGCS1	3-hydroxy-3-methylglutaryl-Coenzyme A synthase 1 (soluble)	6397.65	3761.771	1107.142	9.54E-08	3.91E-06	5
205822_s_at	NM_002130	HMGCS1	3-hydroxy-3-methylglutaryl-Coenzyme A synthase 1 (soluble)	993.113	502.756	92.945	9.88E-08	4.03E-06	5
218898_at	NM_024792	FAM57A	family with sequence similarity 57, member A	690.841	1262.964	613.486	1.01E-07	4.10E-06	17
214734_at	AB014524	EXPH5	exophilin 5	261.836	79.446	176.718	1.03E-07	4.16E-06	11
217173_s_at	S70123	LDLR	low density lipoprotein receptor (familial hypercholesterolemia)	347.264	13.397	29.949	1.03E-07	4.16E-06	19
221577_x_at	BC000529	GDF15	growth differentiation factor 15	218.15	32.722	101.38	1.04E-07	4.16E-06	19
212838_at	AB023227	DNMBP	dynamin binding protein	201	882.55	251.982	1.04E-07	4.17E-06	10
208960_s_at	BE675435	KLF6	Kruppel-like factor 6	546.889	198.199	82.215	1.05E-07	4.19E-06	10
202869_at	NM_016816	OAS1	2',5'-oligoadenylate synthetase 1, 40/46kDa	167.876	74.935	39.71	1.08E-07	4.26E-06	12
207828_s_at	NM_005196	CENPF	centromere protein F,	1514.222	800.311	1656.783	1.08E-07	4.26E-06	1

			350/400ka (mitosin)						
212096_s_at	AL096842	MTUS1	mitochondrial tumor suppressor 1	1028.995	433.463	1017.214	1.10E-07	4.35E-06	8
204794_at	NM_004418	DUSP2	dual specificity phosphatase 2	1177.973	181.785	266.805	1.11E-07	4.36E-06	2
201473_at	NM_002229	JUNB	jun B proto-oncogene	2872.776	685.565	280.487	1.12E-07	4.38E-06	19
209703_x_at	BC004492	METTL7A	methyltransferase like 7A	528.611	342.743	218.559	1.16E-07	4.44E-06	12
201877_s_at	NM_002719	PPP2R5C	protein phosphatase 2, regulatory subunit B', gamma isoform	517.586	248.622	448.574	1.16E-07	4.44E-06	14
207275_s_at	NM_001995	ACSL1	acyl-CoA synthetase long-chain family member 1	97.153	44.71	7.441	1.17E-07	4.48E-06	4
204326_x_at	NM_002450	MT1X	metallothionein 1X	1922.202	19201.9	10991.81	1.18E-07	4.49E-06	16
209631_s_at	U87460	GPR37	G protein-coupled receptor 37 (endothelin receptor type B-like)	568.733	512.045	274.367	1.21E-07	4.55E-06	7
207630_s_at	NM_001881	CREM	cAMP responsive element modulator	1161.015	1050.105	489.509	1.22E-07	4.55E-06	10
219371_s_at	NM_016270	KLF2	Kruppel-like factor 2 (lung)	1281.621	104.839	156.143	1.23E-07	4.59E-06	19
210538_s_at	U37546	BIRC3	baculoviral IAP repeat-containing 3	40.579	146.071	11.783	1.26E-07	4.67E-06	11
202465_at	NM_002593	PCOLCE	procollagen C-endopeptidase enhancer	525.501	428.236	262.475	1.30E-07	4.79E-06	7
205068_s_at	BE671084	ARHGAP26	Rho GTPase activating protein 26	9.335	26.246	11.37	1.34E-07	4.88E-06	5
204580_at	NM_002426	MMP12	matrix metallopeptidase 12 (macrophage elastase)	6.884	335.579	16.295	1.39E-07	5.01E-06	11
222288_at	AI004009			2107.135	1377.497	764.019	1.39E-07	5.01E-06	
213039_at	AB011093	ARHGEF18	rho/rac guanine nucleotide exchange factor (GEF) 18	470.098	1474.882	481.035	1.41E-07	5.04E-06	19
211919_s_at	AF348491	CXCR4	chemokine (C-X-C motif) receptor 4	1249.632	462.692	752.002	1.42E-07	5.07E-06	2
201324_at	NM_001423	EMP1	epithelial membrane protein 1	589.232	1814.536	429.249	1.43E-07	5.09E-06	12
221901_at	BF516072	LL22NC03-75B3.6	KIAA1644 protein	74.497	265.4	112.126	1.44E-07	5.10E-06	22
211456_x_at	AF333388	MT1P2	metallothionein 1 pseudogene 2	1428.759	7527.782	5236.422	1.47E-07	5.17E-06	1
208961_s_at	AB017493	KLF6	Kruppel-like factor 6	1528.976	530.052	233.917	1.47E-07	5.18E-06	10
204476_s_at	NM_022172	PC	pyruvate carboxylase	388.263	384.263	181.711	1.48E-07	5.18E-06	11

202912_at	NM_001124	ADM	adrenomedullin	883.371	2382.73 7	478.547	1.49E-07	5.18E-06	11
202982_s_at	NM_006821	ACOT2	acyl-CoA thioesterase 2	595.002	455.36	272.557	1.54E-07	5.35E-06	14
219959_at	NM_017947	MOCOS	molybdenum cofactor sulfurase	296.6	234.374	596.037	1.56E-07	5.37E-06	18
209239_at	M55643	NFKB1	nuclear factor of kappa light polypeptide gene enhancer in B-cells 1 (p105)	272.363	499.917	220.323	1.57E-07	5.39E-06	4
209605_at	D87292	TST	thiosulfate sulfurtransferase (rhodanese)	1092.575	797.825	523.693	1.62E-07	5.54E-06	22
213309_at	AL117515	PLCL2	phospholipase C-like 2	518.248	809.195	312.604	1.64E-07	5.56E-06	3
201325_s_at	NM_001423	EMP1	epithelial membrane protein 1	118.62	445.63	97.915	1.64E-07	5.56E-06	12
204422_s_at	NM_002006	FGF2	fibroblast growth factor 2 (basic)	728.225	3212.83 2	463.969	1.70E-07	5.74E-06	4
201939_at	NM_006622	PLK2	polo-like kinase 2 (Drosophila)	732.453	1463.83	589.695	1.71E-07	5.75E-06	5
209726_at	AB018195	CA11	carbonic anhydrase XI	107.516	99.998	36.705	1.71E-07	5.75E-06	19
209921_at	AB040875	SLC7A11	solute carrier family 7, (cationic amino acid transporter, y+ system) member 11	86.188	69.651	741.237	1.78E-07	5.94E-06	4
218647_s_at	BE464161	YRDC	yrdC domain containing (E. coli)	626.774	498.433	293.001	1.81E-07	5.99E-06	1
201995_at	NM_000127	EXT1	exostoses (multiple) 1	973.681	1918.92 4	869.046	1.82E-07	6.01E-06	8
208963_x_at	AL512760	FADS1	fatty acid desaturase 1	6056.584	4253.37 3	2121.906	1.88E-07	6.15E-06	11
203242_s_at	BG054550	PDLIM5	PDZ and LIM domain 5	130.845	522.291	135.955	1.89E-07	6.17E-06	4
204015_s_at	BC002671	DUSP4	dual specificity phosphatase 4	222.503	377.594	110.153	1.89E-07	6.17E-06	8
211026_s_at	BC006230	MGLL	monoglyceride lipase	246.107	608.912	350.934	1.95E-07	6.33E-06	3
221873_at	AW162015	ZNF143	zinc finger protein 143	425.116	146.666	173.228	1.98E-07	6.41E-06	11
203286_at	NM_014901	RNF44	ring finger protein 44	140.278	47.846	117.521	2.01E-07	6.49E-06	5
212081_x_at	AF129756	BAT2	HLA-B associated transcript 2	25.491	24.591	59.61	2.08E-07	6.64E-06	6
209012_at	AV718192	TRIO	triple functional domain (PTPRF interacting)	224.938	792.113	278.593	2.11E-07	6.69E-06	5
211423_s_at	D85181	SC5DL	sterol-C5-desaturase (ERG3 delta-5-desaturase homolog, S. cerevisiae)-like	1158.344	779.178	511.05	2.14E-07	6.78E-06	11
210619_s_at	AF173154	HYAL1	hyaluronoglucosaminidase 1	170.943	30.947	95.586	2.17E-07	6.83E-06	3
206170_at	NM_000024	ADRB2	adrenergic, beta-2-, receptor,	270.848	218.801	56.502	2.19E-07	6.89E-06	5

			surface						
218501_at	NM_019555	ARHGEF3	Rho guanine nucleotide exchange factor (GEF) 3	411.208	176.616	354.536	2.37E-07	7.34E-06	3
212070_at	AL554008	GPR56	G protein-coupled receptor 56	250.657	999.938	509.871	2.39E-07	7.37E-06	16
215001_s_at	AL161952	GLUL	glutamate-ammonia ligase (glutamine synthetase)	3829.366	4725.053	1937.794	2.39E-07	7.37E-06	1
218627_at	NM_018370	DRAM	damage-regulated autophagy modulator	1224.935	2238.601	870	2.43E-07	7.44E-06	12
210563_x_at	U97075	CFLAR	CASP8 and FADD-like apoptosis regulator	195.792	388.538	157.39	2.43E-07	7.44E-06	2
201963_at	NM_021122	ACSL1	acyl-CoA synthetase long-chain family member 1	497.18	246.566	59.558	2.46E-07	7.48E-06	4
200797_s_at	AI275690	MCL1	myeloid cell leukemia sequence 1 (BCL2-related)	6737.46	5130.605	2578.646	2.47E-07	7.51E-06	1
209240_at	AF070560	OGT	O-linked N-acetylglucosamine (GlcNAc) transferase (UDP-N-acetylglucosamine:polypeptide-N-acetylglucosaminyl transferase)	1121.571	543.145	1303.214	2.51E-07	7.58E-06	X
208178_x_at	NM_007118	TRIO	triple functional domain (PTPRF interacting)	150.614	415.124	187.364	2.53E-07	7.60E-06	5
200799_at	NM_005345	HSPA1A	heat shock 70kDa protein 1A	7668.11	19395.06	5984.083	2.53E-07	7.60E-06	6
218929_at	NM_017632	CDKN2AIP	CDKN2A interacting protein	616.778	798.886	259.644	2.57E-07	7.68E-06	4
202388_at	NM_002923	RGS2	regulator of G-protein signaling 2, 24kDa	1800.708	2596.102	845.579	2.57E-07	7.68E-06	1
202497_x_at	AI631159	SLC2A3	solute carrier family 2 (facilitated glucose transporter), member 3	856.815	274.175	748.22	2.60E-07	7.72E-06	12
220731_s_at	NM_018090	NECAP2	NECAP endocytosis associated 2	150.988	233.619	107.968	2.67E-07	7.87E-06	1
211981_at	NM_001845	COL4A1	collagen, type IV, alpha 1	48.777	204.003	184.678	2.84E-07	8.21E-06	13
200798_x_at	NM_021960	MCL1	myeloid cell leukemia sequence 1 (BCL2-related)	4867.589	3178.839	1485.66	2.84E-07	8.21E-06	1
219492_at	NM_012110	CHIC2	cysteine-rich hydrophobic domain 2	230.844	360.175	143.312	2.85E-07	8.21E-06	4
218325_s_at	NM_022105	DIDO1	death inducer-obliterator 1	38.975	106.676	71.69	2.86E-07	8.22E-06	20
206461_x_at	NM_005951	MT1H	metallothionein 1H	1276.363	6703.155	4645.276	3.00E-07	8.53E-06	16
218440_at	NM_020166	MCCC1	methylcrotonoyl-Coenzyme A carboxylase 1 (alpha)	139.463	104.85	56.766	3.05E-07	8.65E-06	3

210138_at	AF074979	RGS20	regulator of G-protein signaling 20	81.534	221.801	101.676	3.07E-07	8.69E-06	8
221497_x_at	BC005369	EGLN1	egl nine homolog 1 (C. elegans)	326.866	1042.524	380.869	3.15E-07	8.88E-06	1
213671_s_at	AA621558	MARS	methionyl-tRNA synthetase	1907.297	1968.696	4172.718	3.26E-07	9.11E-06	12
212506_at	AL135735	PICALM	phosphatidylinositol binding clathrin assembly protein	1552.291	3282.407	2021.285	3.26E-07	9.11E-06	11
204178_s_at	NM_006328	RBM14	RNA binding motif protein 14	447.08	1127.348	755.978	3.29E-07	9.18E-06	11
209282_at	AF309082	PRKD2	protein kinase D2	119.003	112.308	256.718	3.37E-07	9.33E-06	19
218284_at	NM_015400	SMAD3	SMAD family member 3	239.682	448.808	137.231	3.39E-07	9.35E-06	15
206374_at	NM_004420	DUSP8	dual specificity phosphatase 8	39.789	5.638	5.443	3.48E-07	9.54E-06	11
200800_s_at	NM_005345	HSPA1A	heat shock 70kDa protein 1A	3909.721	15957.59	2744.418	3.58E-07	9.73E-06	6
202401_s_at	NM_003131	SRF	serum response factor (c-fos serum response element-binding transcription factor)	592.315	596.972	270.475	3.62E-07	9.77E-06	6
202284_s_at	NM_000389	CDKN1A	cyclin-dependent kinase inhibitor 1A (p21, Cip1)	551.544	427.404	217.433	3.62E-07	9.77E-06	6
221985_at	AW006750	KLHL24	kelch-like 24 (Drosophila)	31.964	6.398	8.084	3.63E-07	9.77E-06	3
221979_at	AI150117	TOPORS	topoisomerase I binding, arginine/serine-rich	167.114	158.313	81.767	3.63E-07	9.77E-06	9
212830_at	W68084	MEGF9	multiple EGF-like-domains 9	345.745	69.715	143.586	3.86E-07	1.02E-05	9
206101_at	NM_001393	ECM2	extracellular matrix protein 2, female organ and adipocyte specific	10.23	6.989	72.222	3.96E-07	1.04E-05	9
214508_x_at	U44836	CREM	cAMP responsive element modulator	523.1	442.599	166.484	4.05E-07	1.06E-05	10
221041_s_at	NM_012434	SLC17A5	solute carrier family 17 (anion/sugar transporter), member 5	77.794	24.753	48.734	4.06E-07	1.06E-05	6
203438_at	AI435828	STC2	stanniocalcin 2	12.538	14.294	65.028	4.08E-07	1.06E-05	5
211681_s_at	AF116705	PDLIM5	PDZ and LIM domain 5	36.425	144.397	29.309	4.13E-07	1.07E-05	4
200787_s_at	BC002426	PEA15	phosphoprotein enriched in astrocytes 15	158.03	373.564	297.393	4.14E-07	1.07E-05	1
209682_at	U26710	CBLB	Cas-Br-M (murine) ecotropic retroviral transforming sequence b	38.623	19.048	19.943	4.19E-07	1.08E-05	3
214581_x_at	BE568134	TNFRSF21	tumor necrosis factor receptor superfamily, member 21	25.437	107.444	27.972	4.21E-07	1.09E-05	6

205097_at	AI025519	SLC26A2	solute carrier family 26 (sulfate transporter), member 2	743.368	1652.785	599.395	4.22E-07	1.09E-05	5
201280_s_at	NM_001343	DAB2	disabled homolog 2, mitogen-responsive phosphoprotein (Drosophila)	1623.756	782.91	737.345	4.28E-07	1.10E-05	5
206176_at	NM_001718	BMP6	bone morphogenetic protein 6	703.832	1578.614	1186.386	4.46E-07	1.14E-05	6
209357_at	AF109161	CITED2	Cbp/p300-interacting transactivator, with Glu/Asp-rich carboxy-terminal domain, 2	5164.411	1982.519	1611.903	4.56E-07	1.15E-05	6
208112_x_at	NM_006795	EHD1	EH-domain containing 1	88.381	163.803	81.874	4.57E-07	1.15E-05	11
212201_at	AW274877	ANKLE2	ankyrin repeat and LEM domain containing 2	382.833	856.132	403.207	4.64E-07	1.17E-05	12
218113_at	NM_013390	TMEM2	transmembrane protein 2	95.424	307.965	103.741	4.68E-07	1.17E-05	9
218927_s_at	BC002918	CHST12	carbohydrate (chondroitin 4) sulfotransferase 12	314.291	143.854	155.595	4.76E-07	1.18E-05	7
209636_at	BC002844	NFKB2	nuclear factor of kappa light polypeptide gene enhancer in B-cells 2 (p49/p100)	13.556	37.081	10.766	4.86E-07	1.20E-05	10
207761_s_at	NM_014033	METTL7A	methyltransferase like 7A	3881.372	2575.077	1334.033	4.89E-07	1.20E-05	12
209201_x_at	L01639	CXCR4	chemokine (C-X-C motif) receptor 4	1121.576	366.676	646.94	5.07E-07	1.23E-05	2
201998_at	AI743792	ST6GAL1	ST6 beta-galactosamide alpha-2,6-sialyltranferase 1	315.807	128.957	185.845	5.08E-07	1.23E-05	3
208373_s_at	NM_004154	P2RY6	pyrimidinergic receptor P2Y, G-protein coupled, 6	400.555	217.444	192.851	5.24E-07	1.26E-05	11
201194_at	NM_003009	SEPW1	selenoprotein W, 1	220.839	476.893	673.038	5.29E-07	1.26E-05	19
212462_at	AU144267	MYST4	MYST histone acetyltransferase (monocytic leukemia) 4	217.927	95.301	160.196	5.31E-07	1.26E-05	10
204268_at	NM_005978	S100A2	S100 calcium binding protein A2	59.422	259.663	253.812	5.32E-07	1.26E-05	1
205397_x_at	U76622	SMAD3	SMAD family member 3	20.749	76.253	16.234	5.33E-07	1.26E-05	15
203137_at	NM_004906	WTAP	Wilms tumor 1 associated protein	1449.991	2572.761	1200.072	5.38E-07	1.27E-05	6
213618_at	AB011152	CENTD1	centaurin, delta 1	12.203	37.781	10.341	5.39E-07	1.27E-05	4
212530_at	AL080111	NEK7	NIMA (never in mitosis gene a)-related kinase 7	2570.162	5802.969	2826.5	5.51E-07	1.29E-05	1
213805_at	AI692428	ABHD5	abhydrolase domain	233.94	545.812	218.339	5.57E-07	1.30E-05	3

			containing 5						
203096_s_at	BF439282	RAPGEF2	Rap guanine nucleotide exchange factor (GEF) 2	25.904	63.36	28.469	5.66E-07	1.32E-05	4
217047_s_at	AK027138	FAM13A1	family with sequence similarity 13, member A1	24.782	8.753	31.329	5.79E-07	1.34E-05	4
201133_s_at	AA142966	PJA2	praja 2, RING-H2 motif containing	1084.632	495.639	778.424	5.84E-07	1.35E-05	5
206024_at	NM_002150	HPD	4-hydroxyphenylpyruvate dioxygenase	3465.685	3425.964	1574.468	5.97E-07	1.37E-05	12
209039_x_at	AF001434	EHD1	EH-domain containing 1	76.166	149.806	69.098	6.00E-07	1.37E-05	11
210367_s_at	AF010316	PTGES	prostaglandin E synthase	349.062	613.145	801.669	6.04E-07	1.38E-05	9
218541_s_at	NM_020130	C8orf4	chromosome 8 open reading frame 4	956.749	1535.284	207.633	6.14E-07	1.39E-05	8
212473_s_at	BE965029	MICAL2	microtubule associated monoxygenase, calponin and LIM domain containing 2	223.362	730.813	268.194	6.20E-07	1.40E-05	11
221593_s_at	BC001663	RPL31	ribosomal protein L31	981.364	760.463	446.555	6.32E-07	1.43E-05	2
209288_s_at	AL136842	CDC42EP3	CDC42 effector protein (Rho GTPase binding) 3	329.303	673.752	313.949	6.38E-07	1.44E-05	2
204745_x_at	NM_005950	MT1G	metallothionein 1G	928.728	3512.258	2495.642	6.48E-07	1.45E-05	16
202393_s_at	NM_005655	KLF10	Kruppel-like factor 10	4032.603	2107.614	1366.272	6.52E-07	1.46E-05	8
202289_s_at	NM_006997	TACC2	transforming, acidic coiled-coil containing protein 2	3661.928	2126.638	1394.526	6.54E-07	1.46E-05	10
209868_s_at	D28482	RBMS1	RNA binding motif, single stranded interacting protein 1	369.624	837.842	382.142	6.96E-07	1.53E-05	2
219562_at	NM_014353	RAB26	RAB26, member RAS oncogene family	172.934	104.053	51.527	7.22E-07	1.58E-05	16
219209_at	NM_022168	IFIH1	interferon induced with helicase C domain 1	49.902	21.737	15.047	7.74E-07	1.66E-05	2
205167_s_at	NM_001790	CDC25C	cell division cycle 25 homolog C (S. pombe)	583.781	277.786	349.294	7.81E-07	1.67E-05	5
218943_s_at	NM_014314	DDX58	DEAD (Asp-Glu-Ala-Asp) box polypeptide 58	3195.252	1996.974	1060.503	7.82E-07	1.67E-05	9
205807_s_at	NM_020127	TUFT1	tuftelin 1	842.736	911.124	431.198	7.99E-07	1.70E-05	1
200621_at	NM_004078	CSRP1	cysteine and glycine-rich protein 1	1849.394	4809.751	2474.527	8.26E-07	1.75E-05	1
217122_s_at	AL031282	RP11-345P4.4	similar to solute carrier family 35, member E2	355.677	148.001	197.462	8.78E-07	1.83E-05	1

212907_at	AI972416	SLC30A1	solute carrier family 30 (zinc transporter), member 1	2423.096	7255.15 6	4198.478	8.85E-07	1.84E-05	1
222258_s_at	AF015043	SH3BP4	SH3-domain binding protein 4	313.088	687.143	393.725	8.96E-07	1.85E-05	2
202733_at	NM_004199	P4HA2	procollagen-proline, 2-oxoglutarate 4-dioxygenase (proline 4-hydroxylase), alpha polypeptide II	360.656	682.562	839.202	9.00E-07	1.86E-05	5
221864_at	AW517464	ORAI3	ORAI calcium release-activated calcium modulator 3	34.296	7.056	7.299	9.08E-07	1.86E-05	16
209209_s_at	AW469573	FERMT2	fermitin family homolog 2 (Drosophila)	461.489	1153.89 7	956.156	9.14E-07	1.86E-05	14
211596_s_at	AB050468	LRIG1	leucine-rich repeats and immunoglobulin-like domains 1	49.485	112.251	35.605	9.44E-07	1.91E-05	3
212463_at	BE379006	CD59	CD59 molecule, complement regulatory protein	631.394	889.669	316.758	9.71E-07	1.96E-05	11
222062_at	AI983115	IL27RA	interleukin 27 receptor, alpha	196.722	415.815	222.474	9.89E-07	1.98E-05	19
205798_at	NM_002185	IL7R	interleukin 7 receptor	10.219	51.987	15.604	9.98E-07	1.99E-05	5
209834_at	AB017915	CHST3	carbohydrate (chondroitin 6) sulfotransferase 3	178.076	419.335	248.7	1.00E-06	1.99E-05	10
214771_x_at	AK025604	M-RIP	myosin phosphatase-Rho interacting protein	429.129	792.906	986.063	1.02E-06	2.01E-05	17
205132_at	NM_005159	ACTC1	actin, alpha, cardiac muscle 1	63.983	249.946	74.67	1.02E-06	2.02E-05	15
209305_s_at	AF078077	GADD45B	growth arrest and DNA-damage-inducible, beta	229.292	130.409	53.521	1.03E-06	2.02E-05	19
201127_s_at	NM_001096	ACLY	ATP citrate lyase	2206.28	1662.22 6	1092.501	1.04E-06	2.03E-05	17
216236_s_at	AL110298	SLC2A3	solute carrier family 2 (facilitated glucose transporter), member 3	311.477	108.902	293.412	1.04E-06	2.03E-05	12
38269_at	AL050147	PRKD2	protein kinase D2	129.384	118.336	244.735	1.06E-06	2.04E-05	19
221582_at	BC001193	HIST3H2A	histone cluster 3, H2a	643.202	293.879	387.482	1.06E-06	2.04E-05	1
209011_at	BF223718	TRIO	triple functional domain (PTPRF interacting)	201.478	526.456	277.346	1.07E-06	2.07E-05	5
65438_at	AA195124	KIAA1609	KIAA1609	8.979	22.567	8.944	1.08E-06	2.08E-05	16
208962_s_at	BE540552	FADS1	fatty acid desaturase 1	3946.771	3060.92 6	1572.023	1.10E-06	2.11E-05	11
210512_s_at	AF022375	VEGFA	vascular endothelial growth factor A	2075.64	888.223	1317.029	1.11E-06	2.11E-05	6
202326_at	NM_006709	EHMT2	euchromatic histone-lysine N-methyltransferase 2	101.436	61.959	183.772	1.11E-06	2.12E-05	6

218717_s_at	NM_018192	LEPREL1	leprecan-like 1	377.844	773.818	679.67	1.12E-06	2.13E-05	3
200894_s_at	NM_002014	FKBP4	FK506 binding protein 4, 59kDa	197.294	289.5	488.488	1.13E-06	2.14E-05	12
221737_at	AK024696	GNA12	guanine nucleotide binding protein (G protein) alpha 12	56.234	117.864	56.995	1.19E-06	2.26E-05	7
201169_s_at	BG326045	BHLHB2	basic helix-loop-helix domain containing, class B, 2	661.984	180.783	184.105	1.21E-06	2.28E-05	3
204363_at	NM_001993	F3	coagulation factor III (thromboplastin, tissue factor)	57.949	151.009	51.008	1.22E-06	2.30E-05	1
203153_at	NM_001548	IFIT1	interferon-induced protein with tetratricopeptide repeats 1	9622.064	4762.335	5216.408	1.23E-06	2.31E-05	10
202481_at	NM_004753	DHRS3	dehydrogenase/reductase (SDR family) member 3	83.886	47.977	160.151	1.24E-06	2.31E-05	1
213988_s_at	BE971383	SAT1	spermidine/spermine N1-acetyltransferase 1	443.75	397.282	187.44	1.25E-06	2.34E-05	X
52837_at	AL047020	LL22NC03-75B3.6	KIAA1644 protein	109.946	356.859	146.657	1.27E-06	2.36E-05	22
209210_s_at	Z24725	FERMT2	fermitin family homolog 2 (Drosophila)	2727.395	6115.586	5064.099	1.30E-06	2.41E-05	14
213012_at	D42055	NEDD4	neural precursor cell expressed, developmentally down-regulated 4	312.668	667.605	594.308	1.31E-06	2.42E-05	15
218806_s_at	NM_006113	VAV3	vav 3 guanine nucleotide exchange factor	273.043	16.914	224.994	1.32E-06	2.42E-05	1
218951_s_at	NM_018390	PLCXD1	phosphatidylinositol-specific phospholipase C, X domain containing 1	304.411	156.254	501.713	1.37E-06	2.49E-05	X, Y
203821_at	NM_001945	HBEGF	heparin-binding EGF-like growth factor	40.066	129.213	31.853	1.37E-06	2.49E-05	5
202508_s_at	NM_003081	SNAP25	synaptosomal-associated protein, 25kDa	544.19	417.797	202.025	1.39E-06	2.53E-05	20
205300_s_at	NM_022717	U1SNRNPBP	U11/U12 snRNP 35K	195.846	379.817	170.256	1.41E-06	2.55E-05	12
213330_s_at	BE886580	STIP1	stress-induced-phosphoprotein 1 (Hsp70/Hsp90-organizing protein)	1319.495	1984.526	2906.113	1.41E-06	2.55E-05	11
216218_s_at	AK023546	PLCL2	phospholipase C-like 2	197.217	303.312	138.708	1.41E-06	2.55E-05	3
204285_s_at	NM_021127	PMAIP1	phorbol-12-myristate-13-acetate-induced protein 1	3593.87	1967.459	1372.243	1.44E-06	2.58E-05	18
202498_s_at	BE550486	SLC2A3	solute carrier family 2 (facilitated glucose transporter), member 3	684.124	122.291	432.959	1.45E-06	2.58E-05	12

209505_at	AI951185	NR2F1	nuclear receptor subfamily 2, group F, member 1	608.531	262.928	486.589	1.48E-06	2.63E-05	5
33307_at	AL022316	CTA-126B4.3	CGI-96 protein	91.561	149.455	248.215	1.50E-06	2.66E-05	22
215649_s_at	AF217536	MVK	mevalonate kinase	101.608	16.177	14.993	1.51E-06	2.66E-05	12
206976_s_at	NM_006644	HSPH1	heat shock 105kDa/110kDa protein 1	1039.582	2580.528	1860.61	1.53E-06	2.68E-05	13
219026_s_at	BF435513	RASAL2	RAS protein activator like 2	6.326	29.35	8.016	1.54E-06	2.69E-05	1
202507_s_at	L19760	SNAP25	synaptosomal-associated protein, 25kDa	130.079	120.68	44.776	1.55E-06	2.70E-05	20
212274_at	AV705559	LPIN1	lipin 1	154.526	54.076	70.346	1.56E-06	2.71E-05	2
213116_at	AI191920	NEK3	NIMA (never in mitosis gene a)-related kinase 3	11.629	56.865	53.339	1.57E-06	2.72E-05	13
207980_s_at	NM_006079	CITED2	Cbp/p300-interacting transactivator, with Glu/Asp-rich carboxy-terminal domain, 2	2837.302	1016.346	813.427	1.58E-06	2.74E-05	6
206116_s_at	NM_000366	TPM1	tropomyosin 1 (alpha)	396.767	840.948	523.609	1.58E-06	2.74E-05	15
212248_at	AI886796	MTDH	metadherin	880.062	595.232	1347.645	1.59E-06	2.75E-05	8
218266_s_at	NM_014286	FREQ	frequenin homolog (Drosophila)	55.394	175.451	106.839	1.62E-06	2.78E-05	9
55692_at	W22924	ELMO2	engulfment and cell motility 2	253.883	120.528	218.092	1.63E-06	2.79E-05	20
219534_x_at	NM_000076	CDKN1C	cyclin-dependent kinase inhibitor 1C (p57, Kip2)	209.522	124.777	91.599	1.71E-06	2.88E-05	11
222173_s_at	AK026105	TBC1D2	TBC1 domain family, member 2	49.71	149.318	40.655	1.72E-06	2.89E-05	9
202770_s_at	NM_004354	CCNG2	cyclin G2	213.853	65.64	119.11	1.74E-06	2.92E-05	4
200940_s_at	AI920976	RERE	arginine-glutamic acid dipeptide (RE) repeats	70.637	28.533	63.706	1.75E-06	2.92E-05	1
205168_at	NM_006182	DDR2	discoidin domain receptor tyrosine kinase 2	654.432	355.859	841.087	1.76E-06	2.93E-05	1
212057_at	AA206161	KIAA0182	KIAA0182	540.589	261.837	717.94	1.76E-06	2.93E-05	16
214321_at	BF440025	NOV	nephroblastoma overexpressed gene	1113.673	689.517	1506.206	1.78E-06	2.95E-05	8
78383_at	AI150117	TOPORS	topoisomerase I binding, arginine/serine-rich	94.1	85.54	36.682	1.83E-06	3.00E-05	9
38241_at	U90548	BTN3A3	butyrophilin, subfamily 3, member A3	239.347	76.549	124.175	1.83E-06	3.00E-05	6
218729_at	NM_020169	LXN	latexin	294.475	666.008	420.384	1.83E-06	3.00E-05	3
209048_s_at	AB032951	ZMYND8	zinc finger, MYND-type containing 8	202.912	93.09	190.052	1.87E-06	3.06E-05	20

220707_s_at	NM_024955	FOXRED2	FAD-dependent oxidoreductase domain containing 2	45.26	40.834	95.507	1.88E-06	3.06E-05	22
205631_at	NM_014749	KIAA0586	KIAA0586	35.826	12.726	42.618	1.91E-06	3.11E-05	14
203140_at	NM_001706	BCL6	B-cell CLL/lymphoma 6 (zinc finger protein 51)	421.512	138.367	177.133	1.98E-06	3.20E-05	3
203373_at	NM_003877	SOCS2	suppressor of cytokine signaling 2	331.575	733.678	492.025	2.03E-06	3.26E-05	12
204457_s_at	NM_002048	GAS1	growth arrest-specific 1	6929.43	3301.514	3639.197	2.03E-06	3.26E-05	9
205227_at	NM_002182	IL1RAP	interleukin 1 receptor accessory protein	11.327	34.236	11.717	2.04E-06	3.28E-05	3
203752_s_at	NM_005354	JUND	jun D proto-oncogene	4674.986	1511.79	1792.995	2.05E-06	3.28E-05	19
202859_x_at	NM_000584	IL8	interleukin 8	17.348	8.537	7.286	2.08E-06	3.33E-05	4
204618_s_at	NM_005254	GABPB2	GA binding protein transcription factor, beta subunit 2	476.646	1195.612	651.721	2.15E-06	3.40E-05	15
219694_at	NM_019018	FAM105A	family with sequence similarity 105, member A	122.313	48.841	56.115	2.15E-06	3.40E-05	5
208708_x_at	AL080102	EIF5	eukaryotic translation initiation factor 5	1325.943	2876.492	2907.348	2.33E-06	3.62E-05	14
212095_s_at	BE552421	MTUS1	mitochondrial tumor suppressor 1	110.505	34.617	85.188	2.33E-06	3.62E-05	8
208510_s_at	NM_015869	PPARG	peroxisome proliferator-activated receptor gamma	131.652	267.163	240.359	2.34E-06	3.63E-05	3
219373_at	NM_018973	DPM3	dolichyl-phosphate mannosyltransferase polypeptide 3	1648.733	1462.582	745.106	2.38E-06	3.67E-05	1
204472_at	NM_005261	GEM	GTP binding protein overexpressed in skeletal muscle	58.471	26.766	13.668	2.39E-06	3.68E-05	8
212200_at	AW274877	ANKLE2	ankyrin repeat and LEM domain containing 2	513.026	1220.288	507.21	2.40E-06	3.70E-05	12
213632_at	M94065	DHODH	dihydroorotate dehydrogenase	62.333	49.251	106.015	2.45E-06	3.75E-05	16
201465_s_at	BC002646	JUN	jun oncogene	129.158	24.093	13.151	2.47E-06	3.76E-05	1
221477_s_at	BC001980	MGC5618	hypothetical protein MGC5618	585.464	779.107	324.114	2.48E-06	3.76E-05	
217127_at	AL354872	CTH	cystathionase (cystathionine gamma-lyase)	376.867	260.555	716.967	2.48E-06	3.76E-05	1
221008_s_at	NM_031279	AGXT2L1	alanine-glyoxylate aminotransferase 2-like 1	74.643	24.831	21.282	2.49E-06	3.76E-05	4
210220_at	L37882	FZD2	frizzled homolog 2	276.689	102.222	290.093	2.49E-06	3.76E-05	17

			(Drosophila)						
203910_at	NM_004815	ARHGAP29	Rho GTPase activating protein 29	974.703	1334.428	617.927	2.50E-06	3.77E-05	1
204005_s_at	NM_002583	PAWR	PRKC, apoptosis, WT1, regulator	739.445	1481.214	1079.433	2.52E-06	3.79E-05	12
219825_at	NM_019885	CYP26B1	cytochrome P450, family 26, subfamily B, polypeptide 1	105.713	273.445	110.833	2.52E-06	3.79E-05	2
211924_s_at	AY029180	PLAUR	plasminogen activator, urokinase receptor	420.173	264.245	197.848	2.53E-06	3.80E-05	19
210793_s_at	U41815	NUP98	nucleoporin 98kDa	265.802	508.434	185.998	2.61E-06	3.89E-05	11
202193_at	NM_005569	LIMK2	LIM domain kinase 2	127.804	269.697	158.389	2.61E-06	3.89E-05	22
206549_at	NM_002195	INSL4	insulin-like 4 (placenta)	251.094	498.938	202.826	2.62E-06	3.89E-05	9
218839_at	NM_012258	HEY1	hairy/enhancer-of-split related with YRPW motif 1	151.969	90.704	386.397	2.72E-06	3.99E-05	8
202308_at	NM_004176	SREBF1	sterol regulatory element binding transcription factor 1	645.24	153.263	275.328	2.85E-06	4.16E-05	17
205805_s_at	NM_005012	ROR1	receptor tyrosine kinase-like orphan receptor 1	29.426	105.846	24.454	2.90E-06	4.22E-05	1
205579_at	NM_000861	HRH1	histamine receptor H1	13.345	32.072	11.037	2.92E-06	4.24E-05	3
203628_at	H05812	IGF1R	insulin-like growth factor 1 receptor	2224.467	4373.127	2047.574	2.96E-06	4.27E-05	15
204698_at	NM_002201	ISG20	interferon stimulated exonuclease gene 20kDa	840.174	860.171	367.486	3.02E-06	4.34E-05	15
218426_s_at	BC000787	RNF216	ring finger protein 216	41.087	95.57	110.973	3.07E-06	4.39E-05	7
209674_at	D83702	CRY1	cryptochrome 1 (photolyase-like)	1075.244	2452.506	1188.966	3.10E-06	4.42E-05	12
215714_s_at	AF254822	SMARCA4	SWI/SNF related, matrix associated, actin dependent regulator of chromatin, subfamily a, member 4	697.745	609.028	1236.905	3.11E-06	4.42E-05	19
207574_s_at	NM_015675	GADD45B	growth arrest and DNA-damage-inducible, beta	520.123	287.03	102.088	3.17E-06	4.49E-05	19
218743_at	NM_024591	CHMP6	chromatin modifying protein 6	15.641	45.546	22.944	3.20E-06	4.53E-05	17
219622_at	NM_017817	RAB20	RAB20, member RAS oncogene family	633.87	314.796	453.398	3.25E-06	4.59E-05	13
201565_s_at	NM_002166	ID2	inhibitor of DNA binding 2, dominant negative helix-loop-helix protein	847.041	438.2	1367.504	3.32E-06	4.67E-05	2
205673_s_at	NM_024087	ASB9	ankyrin repeat and SOCS box-containing 9	642.449	493.992	236.477	3.36E-06	4.70E-05	X

207667_s_at	NM_002756	MAP2K3	mitogen-activated protein kinase kinase 3	239.883	568.777	282.393	3.43E-06	4.79E-05	17
202073_at	AV757675	OPTN	optineurin	229.271	203.519	89.332	3.44E-06	4.80E-05	10
211162_x_at	AF116616	SCD	stearoyl-CoA desaturase (delta-9-desaturase)	1635.368	1481.897	326.409	3.48E-06	4.84E-05	10
206665_s_at	NM_001191	BCL2L1	BCL2-like 1	18.215	51.518	18.954	3.52E-06	4.88E-05	20
202769_at	AW134535	CCNG2	cyclin G2	954.908	243.91	441.439	3.64E-06	4.99E-05	4
218280_x_at	BC001629	HIST2H2AA3	histone cluster 2, H2aa3	884.268	933.292	457.425	3.71E-06	5.04E-05	1
209193_at	M24779	PIM1	pim-1 oncogene	77.527	36.017	29.327	3.87E-06	5.20E-05	6
208622_s_at	AA670344	EZR	ezrin	1715.829	3516.568	1874.399	3.89E-06	5.21E-05	6
208763_s_at	AL110191	TSC22D3	TSC22 domain family, member 3	116.893	22.793	71.02	3.91E-06	5.22E-05	X
210090_at	AF193421	ARC	activity-regulated cytoskeleton-associated protein	54.68	4.659	4.621	3.94E-06	5.25E-05	8
204290_s_at	NM_005589	ALDH6A1	aldehyde dehydrogenase 6 family, member A1	81.397	56.095	33.902	4.00E-06	5.31E-05	14
204352_at	NM_004619	TRAF5	TNF receptor-associated factor 5	61.686	123.319	48.604	4.11E-06	5.42E-05	1
220092_s_at	NM_018153	ANTXR1	anthrax toxin receptor 1	355.092	876.361	798.423	4.11E-06	5.42E-05	2
213348_at	N33167	CDKN1C	cyclin-dependent kinase inhibitor 1C (p57, Kip2)	747.125	444.099	274.218	4.20E-06	5.52E-05	11
209260_at	BC000329	SFN	stratifin	883.438	1143.871	458.291	4.21E-06	5.53E-05	1
213164_at	AI867198	SLC5A3	solute carrier family 5 (inositol transporters), member 3	701.863	830.601	348.912	4.24E-06	5.55E-05	21
218543_s_at	NM_022750	PARP12	poly (ADP-ribose) polymerase family, member 12	301.916	472.279	228.97	4.29E-06	5.60E-05	7
202937_x_at	NM_015703	CTA-126B4.3	CGI-96 protein	133.898	199.506	308.215	4.34E-06	5.65E-05	22
218273_s_at	NM_018444	PPM2C	protein phosphatase 2C, magnesium-dependent, catalytic subunit	198.093	213.481	63.948	4.35E-06	5.65E-05	8
204341_at	NM_006470	TRIM16	tripartite motif-containing 16	236.86	600.196	320.041	4.36E-06	5.65E-05	17
216074_x_at	AK001727	WWC1	WW and C2 domain containing 1	50.25	128.378	93.569	4.40E-06	5.70E-05	5
201673_s_at	NM_002103	GYS1	glycogen synthase 1 (muscle)	58.837	60.914	163.816	4.46E-06	5.77E-05	19
36829_at	AF022991	PER1	period homolog 1 (Drosophila)	27.618	10.941	26.445	4.58E-06	5.90E-05	17
204684_at	NM_002522	NPTX1	neuronal pentraxin I	1420.297	3863.813	2101.884	4.60E-06	5.91E-05	17

221986_s_at	AW006750	KLHL24	kelch-like 24 (Drosophila)	39.626	8.563	10.626	4.68E-06	6.00E-05	3
33304_at	U88964	ISG20	interferon stimulated exonuclease gene 20kDa	587.555	582.374	251.61	4.81E-06	6.15E-05	15
204788_s_at	NM_000309	PPOX	protoporphyrinogen oxidase	186.815	170.696	66.946	4.86E-06	6.19E-05	1
213508_at	AA142942	C14orf147	chromosome 14 open reading frame 147	443.62	301.473	202.534	5.03E-06	6.36E-05	14
222016_s_at	AW086021	ZNF323	zinc finger protein 323	137.495	66.417	58.806	5.04E-06	6.36E-05	6
203192_at	NM_005689	ABCB6	ATP-binding cassette, sub-family B (MDR/TAP), member 6	52.856	53.203	122.091	5.05E-06	6.37E-05	2
206377_at	NM_001452	FOXF2	forkhead box F2	64.136	176.174	141.834	5.13E-06	6.45E-05	6
202150_s_at	U64317	NEDD9	neural precursor cell expressed, developmentally down-regulated 9	183.616	318.208	55.214	5.20E-06	6.53E-05	6
222221_x_at	AY007161	EHD1	EH-domain containing 1	67.836	129.894	60.35	5.21E-06	6.54E-05	11
35666_at	U38276	SEMA3F	sema domain, immunoglobulin domain (Ig), short basic domain, secreted, (semaphorin) 3F	279.435	215.16	538.618	5.23E-06	6.55E-05	3
213935_at	AF007132	ABHD5	abhydrolase domain containing 5	77.159	193.959	65.847	5.26E-06	6.57E-05	3
203394_s_at	BE973687	HES1	hairy and enhancer of split 1, (Drosophila)	77.315	33.632	16.627	5.44E-06	6.75E-05	3
209782_s_at	U79283	DBP	D site of albumin promoter (albumin D-box) binding protein	18.216	5.621	31.274	5.59E-06	6.89E-05	19
44040_at	AA524093	FBXO41	F-box protein 41	86.2	82.631	197.848	5.64E-06	6.91E-05	2
203595_s_at	N47725	IFIT5	interferon-induced protein with tetratricopeptide repeats 5	362.01	205.894	75.315	5.66E-06	6.93E-05	10
209846_s_at	BC002832	BTN3A2	butyrophilin, subfamily 3, member A2	598.649	287.42	398.221	5.68E-06	6.94E-05	6
202880_s_at	NM_004762	PSCD1	pleckstrin homology, Sec7 and coiled-coil domains 1(cytohesin 1)	120.189	253.755	134.058	5.74E-06	7.00E-05	17
203414_at	NM_012329	MMD	monocyte to macrophage differentiation-associated	81.797	116.971	52.746	5.90E-06	7.17E-05	17
203395_s_at	NM_005524	HES1	hairy and enhancer of split 1, (Drosophila)	146.657	89.435	71.296	5.91E-06	7.17E-05	3
214828_s_at	AL157851	dJ222E13.2	similar to CGI-96	84.051	179.887	128.379	6.00E-06	7.23E-05	22
211708_s_at	BC005807	SCD	stearoyl-CoA desaturase (delta-9-desaturase)	1430.487	1266.468	348.108	6.07E-06	7.29E-05	10

212565_at	BE302191	STK38L	serine/threonine kinase 38 like	375.894	511.693	196.946	6.25E-06	7.50E-05	12
205157_s_at	NM_000422	KRT17	keratin 17	2973.551	6263.006	1942.471	6.31E-06	7.56E-05	17
216336_x_at	AL031602	MT1M	metallothionein 1M	412.493	1240.084	871.208	6.37E-06	7.62E-05	16
214326_x_at	AI339541	JUND	jun D proto-oncogene	73.436	21.456	26.048	6.37E-06	7.62E-05	19
219195_at	NM_013261	PPARGC1A	peroxisome proliferator-activated receptor gamma, coactivator 1 alpha	111.124	32.707	76.71	6.75E-06	7.98E-05	4
204014_at	NM_001394	DUSP4	dual specificity phosphatase 4	135.687	77.957	23.714	6.77E-06	7.99E-05	8
208903_at	BF431363	RPS28	ribosomal protein S28	344.05	156.05	174.534	6.96E-06	8.16E-05	19
212472_at	BE965029	MICAL2	microtubule associated monoxygenase, calponin and LIM domain containing 2	22.881	91.142	28.691	7.07E-06	8.22E-05	11
204224_s_at	NM_000161	GCH1	GTP cyclohydrolase 1 (dopa-responsive dystonia)	787.134	680.462	349.582	7.21E-06	8.35E-05	14
211139_s_at	AF045452	NAB1	NGFI-A binding protein 1 (EGR1 binding protein 1)	68.994	169.506	66.072	7.27E-06	8.41E-05	2
204985_s_at	NM_024108	TRAPPC6A	trafficking protein particle complex 6A	163.809	149.198	59.461	7.57E-06	8.61E-05	19
218849_s_at	NM_006663	PPP1R13L	protein phosphatase 1, regulatory (inhibitor) subunit 13 like	32.433	115.376	40.741	7.57E-06	8.61E-05	19
214290_s_at	AI313324	HIST2H2AA3	histone cluster 2, H2aa3	3235.443	3365.257	1672.191	7.74E-06	8.77E-05	1
209645_s_at	NM_000692	ALDH1B1	aldehyde dehydrogenase 1 family, member B1	140.499	361.345	105.097	7.82E-06	8.83E-05	9
211382_s_at	AF220152	TACC2	transforming, acidic coiled-coil containing protein 2	950.374	564.751	348.871	7.92E-06	8.91E-05	10
212236_x_at	Z19574	KRT17	keratin 17	3606.601	6640.013	2359.513	8.05E-06	9.02E-05	17
201566_x_at	NM_002166	ID2	inhibitor of DNA binding 2, dominant negative helix-loop-helix protein	76.209	50.685	146.818	8.22E-06	9.13E-05	2
221552_at	BC001698	ABHD6	abhydrolase domain containing 6	81.227	46.667	97.753	8.23E-06	9.13E-05	3
220585_at	NM_025130	HKDC1	hexokinase domain containing 1	17.072	80.327	98.755	8.24E-06	9.13E-05	10
205214_at	NM_004226	STK17B	serine/threonine kinase 17b	29.628	61.399	26.34	8.26E-06	9.13E-05	2
206085_s_at	NM_001902	CTH	cystathionase (cystathionine gamma-lyase)	70.523	46.042	179.115	8.28E-06	9.13E-05	1

203927_at	NM_004556	NFKBIE	nuclear factor of kappa light polypeptide gene enhancer in B-cells inhibitor, epsilon	41.413	71.593	21.529	8.39E-06	9.21E-05	6
205632_s_at	NM_003558	PIP5K1B	phosphatidylinositol-4-phosphate 5-kinase, type I, beta	114.416	17.296	71.726	8.40E-06	9.21E-05	9
204633_s_at	AF074393	RPS6KA5	ribosomal protein S6 kinase, 90kDa, polypeptide 5	178.358	58.638	70.483	8.52E-06	9.31E-05	14
207194_s_at	NM_001544	ICAM4	intercellular adhesion molecule 4 (Landsteiner-Wiener blood group)	70.256	42.348	24.43	8.76E-06	9.49E-05	19
213228_at	AK023913	PDE8B	phosphodiesterase 8B	770.126	302.896	580.143	8.87E-06	9.56E-05	5
212226_s_at	AA628586	PPAP2B	phosphatidic acid phosphatase type 2B	101.127	184.551	84.498	8.95E-06	9.61E-05	1
216607_s_at	U40053	CYP51A1	cytochrome P450, family 51, subfamily A, polypeptide 1	5110.376	4289.972	2178.993	9.01E-06	9.62E-05	7
210971_s_at	AB000815	ARNTL	aryl hydrocarbon receptor nuclear translocator-like	13.31	42.866	15.902	9.10E-06	9.68E-05	11
35436_at	L06147	GOLGA2	golgi autoantigen, golgin subfamily a, 2	333.93	172.968	374.635	9.19E-06	9.74E-05	9
210285_x_at	BC000383	WTAP	Wilms tumor 1 associated protein	192.253	380.856	169.699	9.32E-06	9.87E-05	6
214022_s_at	AA749101	IFITM2	interferon induced transmembrane protein 2 (1-8D)	2804.08	2041.213	1376.807	9.46E-06	9.98E-05	11
218231_at	NM_017567	NAGK	N-acetylglucosamine kinase	154.207	215.912	105.778	9.53E-06	0.0001	2
218901_at	NM_020353	PLSCR4	phospholipid scramblase 4	45.894	16.206	15.487	9.54E-06	0.0001	3
208290_s_at	NM_001969	EIF5	eukaryotic translation initiation factor 5	847.309	2119.125	2101	9.56E-06	0.0001	14
209050_s_at	AI421559	RALGDS	ral guanine nucleotide dissociation stimulator	49.597	21.964	43.92	9.71E-06	0.000101	9
221528_s_at	BC000143	ELMO2	engulfment and cell motility 2	108.333	48.255	99.518	9.72E-06	0.000101	20
203027_s_at	AI189359	MVD	mevalonate (diphospho) decarboxylase	109.696	81.958	19.55	1.01E-05	0.000104	16
202960_s_at	NM_000255	MUT	methylmalonyl Coenzyme A mutase	623.673	370.966	272.105	1.02E-05	0.000105	6
218000_s_at	NM_007350	PHLDA1	pleckstrin homology-like domain, family A, member 1	60.849	103.167	12.398	1.04E-05	0.000106	12
209272_at	AF045451	NAB1	NGFI-A binding protein 1 (EGR1 binding protein 1)	547.597	1023.787	486.637	1.04E-05	0.000106	2

212419_at	AA131324	C10orf56	chromosome 10 open reading frame 56	120.336	59.362	83.139	1.05E-05	0.000106	10
204781_s_at	NM_000043	FAS	Fas (TNF receptor superfamily, member 6)	276.755	282.564	138.266	1.10E-05	0.00011	10
200736_s_at	NM_000581	GPX1	glutathione peroxidase 1	1814.174	3155.032	5118.924	1.14E-05	0.000113	3
202973_x_at	NM_014883	FAM13A1	family with sequence similarity 13, member A1	132.836	49.866	146.589	1.18E-05	0.000116	4
214696_at	AF070569	C17orf91	chromosome 17 open reading frame 91	59.881	95.584	26.051	1.19E-05	0.000117	17
213684_s_at	BF671400	PDLIM5	PDZ and LIM domain 5	26.751	50.12	13.355	1.20E-05	0.000117	4
211122_s_at	AF002985	CXCL11	chemokine (C-X-C motif) ligand 11	47.111	17.08	5.201	1.20E-05	0.000117	4
210827_s_at	U73844	ELF3	E74-like factor 3 (ets domain transcription factor, epithelial-specific)	271.691	134.426	264.209	1.21E-05	0.000118	1
205194_at	NM_004577	PSPH	phosphoserine phosphatase	515.271	363.902	1565.136	1.23E-05	0.000119	7
214960_at	AF229253	API5	apoptosis inhibitor 5	61.707	99.709	147.72	1.24E-05	0.000119	11
219370_at	NM_019845	RPRM	reprimo, TP53 dependent G2 arrest mediator candidate	131.029	58.946	124.429	1.24E-05	0.000119	2
218168_s_at	NM_020247	CABC1	chaperone, ABC1 activity of bc1 complex homolog (S. pombe)	349.981	141.887	231.825	1.24E-05	0.000119	1
205037_at	NM_006860	RABL4	RAB, member of RAS oncogene family-like 4	154.665	130.4	75.815	1.25E-05	0.00012	22
208047_s_at	NM_005966	NAB1	NGFI-A binding protein 1 (EGR1 binding protein 1)	185.689	391.723	182.759	1.30E-05	0.000123	2
201247_at	BE513151	SREBF2	sterol regulatory element binding transcription factor 2	356.749	319.74	150.838	1.30E-05	0.000123	22
217853_at	NM_022748	TNS3	tensin 3	807.279	383.188	556.635	1.33E-05	0.000124	7
219546_at	NM_017593	BMP2K	BMP2 inducible kinase	282.346	517.148	208.137	1.35E-05	0.000126	4
215498_s_at	AA780381	MAP2K3	mitogen-activated protein kinase kinase 3	121.103	378.986	170.659	1.37E-05	0.000127	17
218739_at	NM_016006	ABHD5	abhydrolase domain containing 5	110.481	293.109	177.977	1.39E-05	0.000128	3
217499_x_at	AW874308	OR7E38P	olfactory receptor, family 7, subfamily E, member 38	37.227	45.611	84.191	1.39E-05	0.000129	7

			pseudogene						
218532_s_at	NM_019000	FAM134B	family with sequence similarity 134, member B	208.742	447.023	133.645	1.41E-05	0.00013	5
210524_x_at	AF078844			23.568	77.445	59.445	1.43E-05	0.000131	
212906_at	BE044440	GRAMD1B	GRAM domain containing 1B	236.664	496.113	298.025	1.44E-05	0.000132	11
210117_at	AF311312	SPAG1	sperm associated antigen 1	142.709	401.653	172.35	1.45E-05	0.000132	8
214179_s_at	H93013	NFE2L1	nuclear factor (erythroid-derived 2)-like 1	367.374	266.394	589.27	1.46E-05	0.000133	17
202805_s_at	NM_004996	ABCC1	ATP-binding cassette, sub-family C (CFTR/MRP), member 1	37.292	85.55	77.791	1.46E-05	0.000133	16
222206_s_at	AA781143	NCLN	nicalin homolog (zebrafish)	115.389	144.864	311.104	1.47E-05	0.000134	19
211019_s_at	D63807	LSS	lanosterol synthase (2,3-oxidosqualene-lanosterol cyclase)	37.789	26.353	8.625	1.47E-05	0.000134	21
208947_s_at	U59323	UPF1	UPF1 regulator of nonsense transcripts homolog (yeast)	27.127	37.159	64.681	1.51E-05	0.000136	19
209815_at	BG054916	PTCH1	patched homolog 1 (Drosophila)	437.77	195.653	310.183	1.54E-05	0.000138	9
203748_x_at	NM_016839	RBMS1	RNA binding motif, single stranded interacting protein 1	413.414	813.508	392.102	1.58E-05	0.000141	2
221206_at	NM_024521	PMS2	PMS2 postmeiotic segregation increased 2 (S. cerevisiae)	139.611	72.378	170.123	1.64E-05	0.000145	7
204070_at	NM_004585	RARRES3	retinoic acid receptor responder (tazarotene induced) 3	261.639	199.779	78.556	1.64E-05	0.000145	11
202861_at	NM_002616	PER1	period homolog 1 (Drosophila)	49.584	18.48	45.406	1.64E-05	0.000145	17
32094_at	AB017915	CHST3	carbohydrate (chondroitin 6) sulfotransferase 3	95.515	237.422	128.194	1.65E-05	0.000145	10
204108_at	AL031778	NFYA	nuclear transcription factor Y, alpha	225.268	535.247	280.798	1.68E-05	0.000147	6
204658_at	NM_013293	TRA2A	transformer-2 alpha	383.668	496.883	797.069	1.69E-05	0.000148	7
59644_at	AI735391	BMP2K	BMP2 inducible kinase	244.275	430.484	163.922	1.74E-05	0.000151	4
214283_at	AU150186	IFT20	intraflagellar transport 20 homolog (Chlamydomonas)	423.175	255.262	201.669	1.79E-05	0.000154	17

217025_s_at	AL110225	DBN1	drebrin 1	149.292	148.496	316.669	1.79E-05	0.000154	5
204747_at	NM_001549	IFIT3	interferon-induced protein with tetratricopeptide repeats 3	3423.246	1664.078	1572.195	1.80E-05	0.000155	10
203278_s_at	NM_016621	PHF21A	PHD finger protein 21A	86.791	46.802	101.874	1.83E-05	0.000157	11
212686_at	AB032983	PPM1H	protein phosphatase 1H (PP2C domain containing)	587.67	282.316	434.114	1.84E-05	0.000158	12
202454_s_at	NM_001982	ERBB3	v-erb-b2 erythroblastic leukemia viral oncogene homolog 3 (avian)	20.546	8.947	14.893	1.85E-05	0.000158	12
221269_s_at	NM_031286	SH3BGRL3	SH3 domain binding glutamic acid-rich protein like 3	89.198	136.469	195.834	1.89E-05	0.000161	1
202794_at	NM_002194	INPP1	inositol polyphosphate-1-phosphatase	168.523	288.973	131.57	1.90E-05	0.000162	2
204639_at	NM_000022	ADA	adenosine deaminase	269.321	462.814	543.02	1.90E-05	0.000162	20
205578_at	NM_004560	ROR2	receptor tyrosine kinase-like orphan receptor 2	160.69	78.06	149.542	1.96E-05	0.000166	9
220091_at	NM_017585	SLC2A6	solute carrier family 2 (facilitated glucose transporter), member 6	156.029	108.622	72.964	1.98E-05	0.000166	9
200632_s_at	NM_006096	NDRG1	N-myc downstream regulated gene 1	381.486	797.812	470.776	1.98E-05	0.000166	8
221501_x_at	AF229069	LOC339047	hypothetical protein LOC339047	109.639	127.563	276.325	2.00E-05	0.000168	16
205825_at	NM_000439	PCSK1	proprotein convertase subtilisin/kexin type 1	59.687	27.053	22.772	2.04E-05	0.00017	5
212572_at	AW779556	STK38L	serine/threonine kinase 38 like	664.276	909.013	395.89	2.05E-05	0.000171	12
200895_s_at	NM_002014	FKBP4	FK506 binding protein 4, 59kDa	867.759	1139.581	1751.239	2.06E-05	0.000172	12
213624_at	AA873600	SMPDL3A	sphingomyelin phosphodiesterase, acid-like 3A	213.768	159.823	91.742	2.07E-05	0.000172	6
202581_at	NM_005346	HSPA1B	heat shock 70kDa protein 1B	180.321	888.618	134.814	2.07E-05	0.000172	6
207535_s_at	NM_002502	NFKB2	nuclear factor of kappa light polypeptide gene enhancer in B-cells 2 (p49/p100)	86.074	173.025	43.044	2.13E-05	0.000176	10
206303_s_at	NM_019094	NUDT4	nudix (nucleoside diphosphate linked moiety X)-type motif 4	29.793	37.581	61.97	2.13E-05	0.000176	12

202708_s_at	NM_003528	HIST2H2BE	histone cluster 2, H2be	20.044	35.542	10.213	2.24E-05	0.000183	1
200796_s_at	BF594446	MCL1	myeloid cell leukemia sequence 1 (BCL2-related)	131.281	90.392	33.866	2.24E-05	0.000184	1
218665_at	NM_012193	FZD4	frizzled homolog 4 (Drosophila)	67.306	31.484	36.956	2.27E-05	0.000185	11
210962_s_at	AB019691	AKAP9	A kinase (PRKA) anchor protein (yotiao) 9	454.242	217.488	438.068	2.33E-05	0.000189	7
213785_at	AW269792	IPO9	importin 9	44.527	34.746	20.364	2.38E-05	0.000192	1
207813_s_at	NM_004110	FDXR	ferredoxin reductase	41.567	35.91	18.838	2.39E-05	0.000193	17
208744_x_at	D86956	HSPH1	heat shock 105kDa/110kDa protein 1	596.912	1568.854	1060.772	2.40E-05	0.000193	13
221752_at	AI651213	SSH1	slingshot homolog 1 (Drosophila)	67.256	147.378	73.961	2.45E-05	0.000196	12
218298_s_at	NM_024952	C14orf159	chromosome 14 open reading frame 159	107.337	81.157	48.683	2.49E-05	0.000198	14
207266_x_at	NM_016837	RBMS1	RNA binding motif, single stranded interacting protein 1	373.365	772.271	399.072	2.50E-05	0.000198	2
207214_at	NM_014471	SPINK4	serine peptidase inhibitor, Kazal type 4	317.051	229.629	82.805	2.57E-05	0.000203	9
219094_at	NM_014154	ARMC8	armadillo repeat containing 8	25.698	47.512	17.054	2.63E-05	0.000206	3
208790_s_at	AF312393	PTRF	polymerase I and transcript release factor	354.232	684.093	339.56	2.68E-05	0.000209	17
211792_s_at	U17074	CDKN2C	cyclin-dependent kinase inhibitor 2C (p18, inhibits CDK4)	192.523	96.139	109.278	2.70E-05	0.00021	1
212099_at	AI263909	RHOB	ras homolog gene family, member B	7561.156	3591.792	3617.908	2.71E-05	0.00021	2
207700_s_at	NM_006534	NCOA3	nuclear receptor coactivator 3	734.289	341.608	438.395	2.78E-05	0.000215	20
206332_s_at	NM_005531	IFI16	interferon, gamma-inducible protein 16	968.411	459.27	1001.34	2.78E-05	0.000215	1
38158_at	D79987	ESPL1	extra spindle pole bodies homolog 1 (S. cerevisiae)	219.49	145.515	296.684	2.82E-05	0.000217	12
45288_at	AA209239	ABHD6	abhydrolase domain containing 6	87.04	41.675	82.83	2.83E-05	0.000218	3
204279_at	NM_002800	PSMB9	proteasome (prosome, macropain) subunit, beta type, 9 (large multifunctional	174.021	134.266	55.734	2.85E-05	0.000219	6

			peptidase 2)						
214583_at	AI268381	RSC1A1	regulatory solute carrier protein, family 1, member 1	18.43	37.92	44.458	2.85E-05	0.00021 9	1
204519_s_at	NM_015993	PLLP	plasma membrane proteolipid (plasmolipin)	737.237	321.6	616.548	2.88E-05	0.00022	16
203699_s_at	U53506	DIO2	deiodinase, iodothyronine, type II	164.453	487.274	266.093	2.92E-05	0.00022 2	14
221753_at	AI651213	SSH1	slingshot homolog 1 (Drosophila)	131.45	267.812	128.939	2.93E-05	0.00022 3	12
204817_at	NM_012291	ESPL1	extra spindle pole bodies homolog 1 (S. cerevisiae)	225.142	142.838	310.835	2.94E-05	0.00022 3	12
213119_at	AW058600	SLC36A1	solute carrier family 36 (proton/amino acid symporter), member 1	48.015	104.044	49.915	2.98E-05	0.00022 5	5
219634_at	NM_018413	CHST11	carbohydrate (chondroitin 4) sulfotransferase 11	14.134	53.752	22.681	2.98E-05	0.00022 5	12
201983_s_at	NM_005228	EGFR	epidermal growth factor receptor (erythroblastic leukemia viral (v-erb-b) oncogene homolog, avian)	1531.787	2654.04 2	1233.091	2.99E-05	0.00022 6	7
221588_x_at	AW612403	ALDH6A1	aldehyde dehydrogenase 6 family, member A1	127.004	107.497	43.136	3.17E-05	0.00023 7	14
205046_at	NM_001813	CENPE	centromere protein E, 312kDa	111.249	60.269	127.303	3.22E-05	0.00024	4
213351_s_at	AB018322	TMCC1	transmembrane and coiled-coil domain family 1	82.87	90.511	42.728	3.30E-05	0.00024 5	3
217862_at	NM_016166	PIAS1	protein inhibitor of activated STAT, 1	475.409	217.832	229.754	3.31E-05	0.00024 6	15
204538_x_at	NM_006985	NPIP	nuclear pore complex interacting protein	33.819	39.382	107.537	3.32E-05	0.00024 6	16
200808_s_at	NM_003461	ZYX	zyxin	784.042	2222.07 3	920.844	3.36E-05	0.00024 8	7
213281_at	BE327172	JUN	jun oncogene	39.383	7.696	7.18	3.37E-05	0.00024 9	1
222305_at	AW975638	HK2	hexokinase 2	26.603	19.021	11.793	3.40E-05	0.00025	2
212093_s_at	AI695017	MTUS1	mitochondrial tumor suppressor 1	118.735	60.157	145.463	3.66E-05	0.00026 4	8
201311_s_at	AL515318	SH3BGRL	SH3 domain binding glutamic acid-rich protein like	118.017	96.737	58.751	3.73E-05	0.00026 8	X
205992_s_at	NM_000585	IL15	interleukin 15	423.381	241.934	156.373	3.83E-05	0.00027 4	4

212511_at	AI766247	PICALM	phosphatidylinositol binding clathrin assembly protein	518.395	1140.186	683.212	3.87E-05	0.000276	11
205115_s_at	NM_016196	RBM19	RNA binding motif protein 19	49.141	61.123	103.411	3.90E-05	0.000277	12
218953_s_at	NM_024028	PCYOX1L	prenylcysteine oxidase 1 like	17.441	10.092	6.523	3.90E-05	0.000277	5
205660_at	NM_003733	OASL	2'-5'-oligoadenylate synthetase-like	5662.479	3772.605	2577.882	3.90E-05	0.000277	12
221002_s_at	NM_030927	TSPAN14	tetraspanin 14	22.915	51.62	23.77	3.95E-05	0.000278	10
205231_s_at	NM_005670	EPM2A	epilepsy, progressive myoclonus type 2A, Lafora disease (laforin)	30.11	14.178	14.004	4.01E-05	0.000282	6
209729_at	BC001782	GAS2L1	growth arrest-specific 2 like 1	323.006	148.467	327.922	4.10E-05	0.000287	22
208892_s_at	BC003143	DUSP6	dual specificity phosphatase 6	77.585	37.658	12.733	4.16E-05	0.00029	12
34868_at	AB029012	SMG5	Smg-5 homolog, nonsense mediated mRNA decay factor (C. elegans)	28.658	77.808	67.087	4.21E-05	0.000293	1
204286_s_at	NM_021127	PMAIP1	phorbol-12-myristate-13-acetate-induced protein 1	913.971	602.42	427.658	4.31E-05	0.000298	18
209558_s_at	AB013384	HIP1R	huntingtin interacting protein 1 related	230.926	113.522	142.712	4.34E-05	0.000299	12
202760_s_at	NM_007203	PALM2-AKAP2	PALM2-AKAP2	53.483	133.883	49.448	4.38E-05	0.000301	9
218875_s_at	NM_012177	FBXO5	F-box protein 5	491.913	1074.981	740.555	4.39E-05	0.000301	6
203339_at	AI887457	SLC25A12	solute carrier family 25 (mitochondrial carrier, Aralar), member 12	139.683	59.39	144.25	4.42E-05	0.000303	2
207760_s_at	NM_006312	NCOR2	nuclear receptor co-repressor 2	679.481	529.625	1082.866	4.44E-05	0.000303	12
209016_s_at	BC002700	KRT7	keratin 7	2645.989	5328.814	5178.436	4.46E-05	0.000304	12
205552_s_at	NM_002534	OAS1	2',5'-oligoadenylate synthetase 1, 40/46kDa	278.888	199.81	135.275	4.49E-05	0.000305	12
214444_s_at	NM_006505	PVR	poliovirus receptor	27.332	73.406	49.207	4.50E-05	0.000306	19
204501_at	NM_002514	NOV	nephroblastoma overexpressed gene	411.467	245.671	548.728	4.58E-05	0.00031	8
209005_at	AF157323	FBXL5	F-box and leucine-rich repeat protein 5	114.798	161.465	75.613	4.75E-05	0.000318	4

220648_at	NM_018702	ADARB2	adenosine deaminase, RNA-specific, B2 (RED2 homolog rat)	17.539	18.469	61.015	4.89E-05	0.000326	10
213030_s_at	AI688418	PLXNA2	plexin A2	14.991	56.915	16.841	4.97E-05	0.00033	1
201340_s_at	AF010314	ENC1	ectodermal-neural cortex (with BTB-like domain)	68.486	47.94	19.92	4.98E-05	0.000331	5
36564_at	W27419	RNF19B	ring finger protein 19B	12.261	23.315	10.192	5.03E-05	0.000333	1
212692_s_at	W60686	LRBA	LPS-responsive vesicle trafficking, beach and anchor containing	426.178	352.126	200.42	5.03E-05	0.000333	4
213182_x_at	R78668	CDKN1C	cyclin-dependent kinase inhibitor 1C (p57, Kip2)	229.309	115.882	83.287	5.07E-05	0.000335	11
208965_s_at	BG256677	IFI16	interferon, gamma-inducible protein 16	330.883	125.267	270.715	5.08E-05	0.000335	1
221883_at	AA133342	PKNOX1	PBX/knotted 1 homeobox 1	46.243	83.243	39.022	5.08E-05	0.000335	21
219437_s_at	NM_013275	ANKRD11	ankyrin repeat domain 11	69.738	54.15	176.551	5.23E-05	0.000344	16
214446_at	NM_012081	ELL2	elongation factor, RNA polymerase II, 2	70.139	227.62	111.266	5.26E-05	0.000345	5
202759_s_at	BE879367	AKAP2	A kinase (PRKA) anchor protein 2	49.566	116.065	38.686	5.27E-05	0.000346	9
210797_s_at	AF063612	OASL	2'-5'-oligoadenylate synthetase-like	6356.583	4377.251	3006.344	5.32E-05	0.000348	12
214755_at	AK022632	UAP1L1	UDP-N-acteylglucosamine pyrophosphorylase 1-like 1	54.54	51.49	110.578	5.38E-05	0.00035	9
216870_x_at	AF264787	DLEU2	deleted in lymphocytic leukemia, 2	18.863	44.318	42.816	5.38E-05	0.00035	13
208712_at	M73554	CCND1	cyclin D1	150.261	322.82	280.91	5.62E-05	0.000361	11
211168_s_at	D86988	UPF1	UPF1 regulator of nonsense transcripts homolog (yeast)	92.039	150.746	195.763	5.66E-05	0.000364	19
201208_s_at	NM_021137	TNFAIP1	tumor necrosis factor, alpha-induced protein 1 (endothelial)	300.858	564.726	231.694	5.73E-05	0.000367	17
212298_at	BE620457	NRP1	neuropilin 1	119.489	53.414	90.078	5.74E-05	0.000368	10
221250_s_at	NM_031300	MXD3	MAX dimerization protein 3	114.206	46.901	56.015	5.87E-05	0.000375	5
213929_at	AL050204			101.393	25.415	66.022	5.92E-05	0.000376	
212218_s_at	AI954041	FASN	fatty acid synthase	1523.221	1383.89	446.29	5.94E-05	0.00037	17

					5			7	
202103_at	AI991631	BRD4	bromodomain containing 4	35.28	72.713	57.996	5.99E-05	0.00038	19
218248_at	NM_022074	FAM111A	family with sequence similarity 111, member A	76.665	46.275	36.942	6.15E-05	0.000386	11
208926_at	U84246	NEU1	sialidase 1 (lysosomal sialidase)	98.549	78.912	47.749	6.20E-05	0.000388	6
218764_at	NM_024064	PRKCH	protein kinase C, eta	5.246	22.294	5.249	6.25E-05	0.000391	14
203787_at	NM_012446	SSBP2	single-stranded DNA binding protein 2	239.316	125.247	111.068	6.26E-05	0.000391	5
218858_at	NM_022783	DEPDC6	DEP domain containing 6	92.623	30.886	28.029	6.32E-05	0.000393	8
203939_at	NM_002526	NT5E	5'-nucleotidase, ecto (CD73)	114.945	257.344	266.684	6.33E-05	0.000394	6
201389_at	NM_002205	ITGA5	integrin, alpha 5 (fibronectin receptor, alpha polypeptide)	144.308	338.554	283.332	6.39E-05	0.000397	12
212662_at	BE615277	PVR	poliovirus receptor	161.849	426.182	271.304	6.50E-05	0.000402	19
215037_s_at	U72398	BCL2L1	BCL2-like 1	11.112	28.099	11.24	6.50E-05	0.000402	20
206155_at	NM_000392	ABCC2	ATP-binding cassette, sub-family C (CFTR/MRP), member 2	100.188	92.902	195.691	6.51E-05	0.000402	10
212944_at	AK024896	SLC5A3	solute carrier family 5 (inositol transporters), member 3	408.89	464.164	200.734	6.53E-05	0.000403	21
214035_x_at	AA308853	LOC399491	LOC399491 protein	34.171	38.974	82.931	6.53E-05	0.000403	16
211559_s_at	L49506	CCNG2	cyclin G2	86.192	15.302	39.71	6.64E-05	0.000408	4
211962_s_at	BG250310	ZFP36L1	zinc finger protein 36, C3H type-like 1	1314.836	632.434	542.296	6.68E-05	0.00041	14
204855_at	NM_002639	SERPINB5	serpin peptidase inhibitor, clade B (ovalbumin), member 5	114.808	171.145	230.724	6.92E-05	0.000421	18
208710_s_at	AI424923	AP3D1	adaptor-related protein complex 3, delta 1 subunit	113.35	72.647	146.561	7.13E-05	0.00043	19
212022_s_at	AU132185	MKI67	antigen identified by monoclonal antibody Ki-67	343.35	317.337	704.569	7.22E-05	0.000434	10
221895_at	AW469184	MOSPD2	motile sperm domain containing 2	134.459	49.85	95.513	7.22E-05	0.000434	X
213457_at	BF739959	MFHAS1	malignant fibrous histiocytoma amplified sequence 1	46.698	82.369	33.484	7.26E-05	0.000436	8

205014_at	NM_005130	FGFBP1	fibroblast growth factor binding protein 1	57.785	46.667	26.818	7.33E-05	0.000438	4
221903_s_at	BF516433	CYLD	cylindromatosis (turban tumor syndrome)	24.522	39.701	14.915	7.48E-05	0.000445	16
209904_at	AF020769	TNNC1	troponin C type 1 (slow)	162.561	172.008	81.459	7.50E-05	0.000445	3
209037_s_at	AW182860	EHD1	EH-domain containing 1	147.132	329.673	170.792	7.63E-05	0.000452	11
200962_at	AI348010	RPL31	ribosomal protein L31	290.998	146.263	61.986	7.72E-05	0.000456	2
203592_s_at	NM_005860	FSTL3	follistatin-like 3 (secreted glycoprotein)	330.457	595.594	692.18	7.73E-05	0.000456	19
215078_at	AL050388	SOD2	superoxide dismutase 2, mitochondrial	29.49	14.94	6.059	7.95E-05	0.000465	6
209355_s_at	AB000889	PPAP2B	phosphatidic acid phosphatase type 2B	98.803	214.321	91	8.06E-05	0.000471	1
217785_s_at	NM_006555	YKT6	YKT6 v-SNARE homolog (S. cerevisiae)	47.475	71.809	111.43	8.17E-05	0.000475	7
204167_at	NM_000060	BTD	biotinidase	69.098	129.562	49.155	8.25E-05	0.000479	3
219221_at	NM_024724	ZBTB38	zinc finger and BTB domain containing 38	983.937	2014.947	1072.133	8.35E-05	0.000483	3
218401_s_at	AU150752	ZNF281	zinc finger protein 281	147.147	84.233	69.848	8.43E-05	0.000486	1
206752_s_at	NM_004402	DFFB	DNA fragmentation factor, 40kDa, beta polypeptide (caspase-activated DNase)	33.284	14.32	31.316	8.49E-05	0.000489	1
203986_at	NM_003943	STBD1	starch binding domain 1	19.108	37.907	17.055	8.55E-05	0.000492	4
205619_s_at	NM_004527	MEOX1	mesenchyme homeobox 1	93.73	40.357	28.386	8.59E-05	0.000493	17
202411_at	NM_005532	IFI27	interferon, alpha-inducible protein 27	151.108	115.884	40.564	8.67E-05	0.000496	14
202637_s_at	AI608725	ICAM1	intercellular adhesion molecule 1 (CD54), human rhinovirus receptor	120.4	265.182	181.485	8.67E-05	0.000496	19
217502_at	BE888744	IFIT2	interferon-induced protein with tetratricopeptide repeats 2	765.641	274.973	327.489	8.68E-05	0.000496	10
212822_at	AA121502	HEG1	HEG homolog 1 (zebrafish)	182.45	413.925	318.275	8.78E-05	0.000501	3
205773_at	NM_014912	CPEB3	cytoplasmic polyadenylation element binding protein 3	52.563	21.885	29.523	8.81E-05	0.000502	10

215236_s_at	AV721177	PICALM	phosphatidylinositol binding clathrin assembly protein	255.956	693.439	373.948	8.83E-05	0.000503	11
203491_s_at	AI123527	CEP57	centrosomal protein 57kDa	1081.329	492.266	658.275	8.99E-05	0.000509	11
205698_s_at	U39657	MAP2K6	mitogen-activated protein kinase kinase 6	251.78	115.563	163.405	9.54E-05	0.000531	17
204838_s_at	NM_014381	MLH3	mutL homolog 3 (E. coli)	11.715	14.704	23.542	9.62E-05	0.000534	14
209379_s_at	AF241785	KIAA1128	KIAA1128	92.182	64.429	42.171	9.75E-05	0.00054	10
207388_s_at	NM_004878	PTGES	prostaglandin E synthase	108.895	255.468	372.646	9.81E-05	0.000543	9
217591_at	BF725121	SKIL	SKI-like oncogene	130.794	82.952	46.209	9.85E-05	0.000544	3
217150_s_at	S73854	NF2	neurofibromin 2 (merlin)	67.487	99.095	144.007	9.90E-05	0.000546	22
205027_s_at	NM_005204	MAP3K8	mitogen-activated protein kinase kinase kinase 8	87.65	39.249	26.781	0.000103	0.000562	10
202969_at	AI216690			98.091	205.851	117.487	0.000103	0.000562	
202127_at	AB011108	PRPF4B	PRP4 pre-mRNA processing factor 4 homolog B (yeast)	38.533	53.984	84.268	0.000103	0.000562	6
213675_at	W61005			34.643	38.627	16.57	0.000104	0.000564	
214045_at	BF056778	LIAS	lipoic acid synthetase	55.085	39.363	90.164	0.000106	0.000572	4
206832_s_at	NM_004186	SEMA3F	sema domain, immunoglobulin domain (Ig), short basic domain, secreted, (semaphorin) 3F	5.039	4.667	16.153	0.000108	0.000579	3
210086_at	AF039196	HR	hairless homolog (mouse)	8.149	7.745	21.194	0.000109	0.000583	8
204136_at	NM_000094	COL7A1	collagen, type VII, alpha 1 (epidermolysis bullosa, dystrophic, dominant and recessive)	10.594	23.965	24.988	0.000111	0.000593	3
203372_s_at	NM_003877	SOCS2	suppressor of cytokine signaling 2	59.76	171.492	108.2	0.000112	0.000596	12
220520_s_at	NM_017681	NUP62CL	nucleoporin 62kDa C-terminal like	57.185	97.971	34.906	0.000113	0.0006	X
211977_at	AK024651	GPR107	G protein-coupled receptor 107	29.864	60.07	49.368	0.000116	0.000613	9
204032_at	NM_003567	BCAR3	breast cancer anti-estrogen	266.256	500.902	248.896	0.00012	0.00063	1

			resistance 3				1	5	
210612_s_at	AF318616	SYNJ2	synaptojanin 2	128.961	242.721	118.057	0.00012 7	0.00066	6
203780_at	AF275945	MPZL2	myelin protein zero-like 2	84.161	242.071	76.729	0.00013	0.00067 2	11
204435_at	NM_014778	NUPL1	nucleoporin like 1	154.409	173.155	69.482	0.00013 6	0.00069 7	13
218880_at	NM_024530	FOSL2	FOS-like antigen 2	119.207	160.355	79.218	0.00013 9	0.00070 8	2
214061_at	AI017564	WDR67	WD repeat domain 67	93.799	52.87	107.631	0.00014	0.00071 2	8
213327_s_at	AI820101	USP12	ubiquitin specific peptidase 12	472.632	1010.77	649.441	0.00014	0.00071 3	13
205401_at	NM_003659	AGPS	alkylglycerone phosphate synthase	197.885	349.006	405.266	0.00014 5	0.00072 8	2
214870_x_at	AC002045	LOC339047	hypothetical protein LOC339047	44.519	59.571	128.258	0.00014 9	0.00074 4	16
215706_x_at	BC002323	ZYX	zyxin	417.243	1283.00 7	545.572	0.00015 1	0.00075 2	7
203494_s_at	NM_014679	CEP57	centrosomal protein 57kDa	1165.183	529.693	697.955	0.00015 5	0.00076 6	11
216049_at	AK023621	RHOBTB3	Rho-related BTB domain containing 3	12.502	30.736	14.203	0.00015 7	0.00077 4	5
209003_at	AF070548	SLC25A11	solute carrier family 25 (mitochondrial carrier; oxoglutarate carrier), member 11	125.976	165.078	262.743	0.00015 8	0.00077 7	17
221122_at	NM_017878	HRASLS2	HRAS-like suppressor 2	45.737	31.524	13.277	0.00016 3	0.00079 5	11
206240_s_at	NM_003437	ZNF136	zinc finger protein 136	217.193	132.381	104.755	0.00016 4	0.0008	19
206544_x_at	NM_003070	SMARCA2	SWI/SNF related, matrix associated, actin dependent regulator of chromatin, subfamily a, member 2	152.98	66.993	162.007	0.00016 8	0.00081 3	9
213397_x_at	AI761728	ANG	angiogenin, ribonuclease, RNase A family, 5	17.099	11.709	7.365	0.00016 9	0.00081 6	14
218818_at	NM_004468	FHL3	four and a half LIM domains 3	12.139	25.552	14.234	0.00016 9	0.00081 7	1
220123_at	NM_025181	SLC35F5	solute carrier family 35, member F5	13.113	22.785	9.647	0.00017	0.00081 8	2

203455_s_at	NM_002970	SAT1	spermidine/spermine N1-acetyltransferase 1	744.53	923.939	454.102	0.000173	0.000831	X
221510_s_at	AI828035	GLS	glutaminase	714.186	1440.287	831.216	0.000189	0.000886	2
220177_s_at	NM_024022	TMPRSS3	transmembrane protease, serine 3	113.942	71.606	49.549	0.000191	0.000895	21
204011_at	NM_005842	SPRY2	sprouty homolog 2 (Drosophila)	315.871	263.457	139.242	0.000192	0.000897	13
212343_at	AL117461	YIPF6	Yip1 domain family, member 6	90.617	107.9	52.967	0.000192	0.000899	X
206907_at	NM_003811	TNFSF9	tumor necrosis factor (ligand) superfamily, member 9	566.78	275.25	501.707	0.000197	0.00092	19
219235_s_at	NM_023923	PHACTR4	phosphatase and actin regulator 4	32.971	19.579	49.608	0.000199	0.000926	1
201101_s_at	BE963370	BCLAF1	BCL2-associated transcription factor 1	402.897	364.057	765.599	0.000204	0.000943	6
217202_s_at	U08626	GLUL	glutamate-ammonia ligase (glutamine synthetase)	422.536	709.268	231.655	0.000204	0.000944	1
209340_at	S73498	UAP1	UDP-N-acteylglucosamine pyrophosphorylase 1	3027.253	6154.513	3660.237	0.000204	0.000946	1
217992_s_at	AW664179	EFHD2	EF-hand domain family, member D2	234.227	554.082	271.925	0.000206	0.000949	1
220658_s_at	NM_020183	ARNTL2	aryl hydrocarbon receptor nuclear translocator-like 2	32.192	71.747	33.111	0.00021	0.000967	12
219523_s_at	NM_018104	ODZ3	odz, odd Oz/ten-m homolog 3 (Drosophila)	52.782	26.389	68.695	0.000211	0.000967	4
209587_at	U70370	PITX1	paired-like homeodomain 1	32.529	27.183	60.749	0.000212	0.00097	5
220917_s_at	NM_025132	WDR19	WD repeat domain 19	129.254	58.295	93.892	0.000213	0.000976	4
204804_at	NM_003141	TRIM21	tripartite motif-containing 21	29.217	41.712	18.114	0.000214	0.00098	11
214104_at	AI703188	GPR161	G protein-coupled receptor 161	150.014	175.61	69.618	0.000215	0.000982	1
211814_s_at	AF112857	CCNE2	cyclin E2	168.047	361.898	164.536	0.000234	0.001051	8
203230_at	AF006011	DVL1	dishevelled, dsh homolog 1 (Drosophila)	50.771	39.461	85.9	0.000238	0.001066	1
215253_s_at	AL049369	RCAN1	regulator of calcineurin 1	285.651	145.746	110.405	0.000239	0.001069	21
202321_at	AW299507	GGPS1	geranylgeranyl diphosphate synthase 1	76.845	28.28	26.442	0.00024	0.001072	1

200648_s_at	NM_002065	GLUL	glutamate-ammonia ligase (glutamine synthetase)	484.859	820.755	271.092	0.000256	0.001127	1
204361_s_at	AB014486	SKAP2	src kinase associated phosphoprotein 2	49.963	28.944	20.808	0.000258	0.001134	7
201043_s_at	NM_006305	ANP32A	acidic (leucine-rich) nuclear phosphoprotein 32 family, member A	508.281	553.986	1175.139	0.00026	0.001139	15
201341_at	NM_003633	ENC1	ectodermal-neural cortex (with BTB-like domain)	35.3	39.653	19.807	0.00026	0.001141	5
205203_at	NM_002662	PLD1	phospholipase D1, phosphatidylcholine-specific	61.53	27.741	44.563	0.00027	0.001174	3
201702_s_at	AI492873	PPP1R10	protein phosphatase 1, regulatory (inhibitor) subunit 10	140.352	42.824	56.105	0.000271	0.001178	6
210387_at	BC001131	HIST1H2BG	histone cluster 1, H2bg	169.555	129.134	48.05	0.000287	0.001235	6
212388_at	AB028980	USP24	ubiquitin specific peptidase 24	48.787	78.923	128.6	0.000298	0.001271	1
64408_s_at	AW025529	CALML4	calmodulin-like 4	13.944	12.438	26.194	0.000299	0.001275	15
206582_s_at	NM_005682	GPR56	G protein-coupled receptor 56	9.243	48.085	17.264	0.000306	0.0013	16
214496_x_at	NM_012330	MYST4	MYST histone acetyltransferase (monocytic leukemia) 4	28.232	12.74	16.304	0.000309	0.001309	10
215287_at	AA975427			44	24.274	20.682	0.00031	0.001309	
209877_at	AF010126	SNCG	synuclein, gamma (breast cancer-specific protein 1)	125.876	137.923	67.59	0.00031	0.00131	10
209360_s_at	BF432501	RUNX1	runt-related transcription factor 1 (acute myeloid leukemia 1; aml1 oncogene)	56.481	127.708	54.216	0.00032	0.001342	21
218177_at	NM_020412	CHMP1B	chromatin modifying protein 1B	143.976	60.55	71.379	0.000327	0.001367	18
215629_s_at	AA905286	DLEU2L	deleted in lymphocytic leukemia 2-like	41.213	118.114	116.506	0.000328	0.00137	1
216863_s_at	AC004542	MORC2	MORC family CW-type zinc finger 2	59.655	75.668	120.63	0.000329	0.001372	22
206079_at	NM_001821	CHML	choroideremia-like (Rab escort protein 2)	15.721	34.702	17.714	0.000339	0.001404	1
215033_at	AI189753	TM4SF1	transmembrane 4 L six family member 1	18.61	17.13	7.771	0.000341	0.001407	3

219956_at	NM_007210	GALNT6	UDP-N-acetyl-alpha-D-galactosamine:polypeptide N-acetylgalactosaminyltransferase 6 (GalNAc-T6)	72.082	48.617	99.045	0.000352	0.001444	12
218500_at	NM_016647	C8orf55	chromosome 8 open reading frame 55	89.993	44.23	65.2	0.000353	0.001444	8
218483_s_at	NM_020153	C11orf60	chromosome 11 open reading frame 60	50.484	99.285	110.223	0.000353	0.001445	11
201058_s_at	NM_006097	MYL9	myosin, light chain 9, regulatory	206.679	441.525	363.931	0.000358	0.001462	20
203946_s_at	NM_001172	ARG2	arginase, type II	34.457	20.548	13.404	0.000377	0.001531	14
177_at	U38545	PLD1	phospholipase D1, phosphatidylcholine-specific	25.435	11.276	16.016	0.000381	0.00154	3
206451_at	NM_018138	TBCCD1	TBCC domain containing 1	231.16	359.905	173.997	0.000382	0.001543	3
212009_s_at	AL553320	STIP1	stress-induced-phosphoprotein 1 (Hsp70/Hsp90-organizing protein)	648.965	1124.852	1555.465	0.000386	0.001555	11
203349_s_at	NM_004454	ETV5	ets variant gene 5 (ets-related molecule)	150.296	174.958	86.514	0.000395	0.001583	3
204039_at	NM_004364	CEBPA	CCAAT/enhancer binding protein (C/EBP), alpha	24.196	16.55	35.23	0.000401	0.001602	19
221088_s_at	NM_017650	PPP1R9A	protein phosphatase 1, regulatory (inhibitor) subunit 9A	78.703	23.898	37.153	0.000416	0.00165	7
211317_s_at	AF041461	CFLAR	CASP8 and FADD-like apoptosis regulator	9.479	26.358	8.977	0.000419	0.001661	2
203661_s_at	NM_003275	TMOD1	tropomodulin 1	48.4	41.523	18.101	0.000421	0.001664	9
214602_at	D17391	COL4A4	collagen, type IV, alpha 4	41.895	23.301	19.868	0.000423	0.001671	2
202630_at	AA046411	APPBP2	amyloid beta precursor protein (cytoplasmic tail) binding protein 2	130.205	69.608	52.075	0.000427	0.001682	17
210530_s_at	M21985	NR2C1	nuclear receptor subfamily 2, group C, member 1	53.788	116.703	97.074	0.00043	0.001692	12
209784_s_at	AF029778	JAG2	jagged 2	107.377	84.987	180.957	0.000442	0.001731	14
203370_s_at	NM_005451	PDLIM7	PDZ and LIM domain 7 (enigma)	49.643	133.276	92.971	0.000447	0.001745	5

209351_at	BC002690	KRT14	keratin 14 (epidermolysis bullosa simplex, Dowling-Meara, Koebner)	15.242	70.361	36.997	0.000451	0.001755	17
206833_s_at	NM_001108	ACYP2	acylphosphatase 2, muscle type	125.086	121.903	61.567	0.000451	0.001755	2
202638_s_at	NM_000201	ICAM1	intercellular adhesion molecule 1 (CD54), human rhinovirus receptor	125.85	251.702	154.385	0.000458	0.001773	19
203080_s_at	NM_013450	BAZ2B	bromodomain adjacent to zinc finger domain, 2B	42.449	18.265	34.162	0.000463	0.001787	2
207020_at	NM_007031	HSF2BP	heat shock transcription factor 2 binding protein	37.407	96.72	46.922	0.000463	0.001787	21
212256_at	BE906572	GALNT10	UDP-N-acetyl-alpha-D-galactosamine:polypeptide N-acetylgalactosaminyltransferase 10 (GalNAc-T10)	35.063	73.481	52.172	0.000473	0.00182	5
202692_s_at	NM_014233	UBTF	upstream binding transcription factor, RNA polymerase I	55.941	89.776	115.168	0.00048	0.001842	17
203596_s_at	NM_012420	IFIT5	interferon-induced protein with tetratricopeptide repeats 5	23.05	8.032	6.966	0.000484	0.001854	10
213548_s_at	BG257762	CDV3	CDV3 homolog (mouse)	212.764	470.384	252.482	0.000485	0.001856	3
210675_s_at	U77917	PTPRR	protein tyrosine phosphatase, receptor type, R	63.446	34.893	97.273	0.000496	0.001885	12
205547_s_at	NM_003186	TAGLN	transgelin	10.388	48.525	28.82	0.00051	0.001927	11
220187_at	NM_024636	STEAP4	STEAP family member 4	27.855	26.598	13.211	0.00052	0.001959	7
203643_at	NM_006494	ERF	Ets2 repressor factor	37.035	33.056	72.18	0.000531	0.001992	19
214266_s_at	AW206786	PDLIM7	PDZ and LIM domain 7 (enigma)	17.483	50.026	39.339	0.000531	0.001994	5
213546_at	AL050378	DKFZp586I1420	hypothetical protein DKFZp586I1420	37.689	35.326	17.322	0.000533	0.001998	7
219822_at	NM_004294	MTRF1	mitochondrial translational release factor 1	38.798	16.772	24.011	0.000535	0.002007	13
203059_s_at	NM_004670	PAPSS2	3'-phosphoadenosine 5'-phosphosulfate synthase 2	46.195	65.053	110.394	0.000618	0.002251	10
218502_s_at	NM_014112	TRPS1	trichorhinophalangeal syndrome I	120.782	56.281	100.992	0.000622	0.002263	8
217234_s_at	AF199015	EZR	ezrin	956.752	2141.754	1089.242	0.000625	0.002272	6

206084_at	NM_002849	PTPRR	protein tyrosine phosphatase, receptor type, R	120.845	56.469	135.269	0.000627	0.002276	12
214056_at	BF981280	MCL1	myeloid cell leukemia sequence 1 (BCL2-related)	199.456	68.254	47.943	0.000639	0.002312	1
213668_s_at	AI989477	SOX4	SRY (sex determining region Y)-box 4	16.248	37.395	18.852	0.000662	0.002372	6
208621_s_at	BF663141	EZR	ezrin	694.541	1672.084	785.718	0.000667	0.00238	6
202314_at	NM_000786	CYP51A1	cytochrome P450, family 51, subfamily A, polypeptide 1	7809.224	6177.213	3845.958	0.000677	0.002409	7
206578_at	NM_004387	NKX2-5	NK2 transcription factor related, locus 5 (Drosophila)	57.667	35.145	25.229	0.000691	0.002448	5
203860_at	NM_000282	PCCA	propionyl Coenzyme A carboxylase, alpha polypeptide	155.038	135.268	76.723	0.000704	0.002481	13
213669_at	AB006628	FCHO1	FCH domain only 1	20.865	12.152	24.795	0.000711	0.002502	19
204107_at	BF445142	NFYA	nuclear transcription factor Y, alpha	87.839	177.661	107.897	0.000752	0.002614	6
210346_s_at	AF212224	CLK4	CDC-like kinase 4	53.384	69.809	29.9	0.000758	0.002629	5
204466_s_at	NM_000345	SNCA	synuclein, alpha (non A4 component of amyloid precursor)	172.277	78.696	112.372	0.000773	0.002667	4
205396_at	BF971416	SMAD3	SMAD family member 3	6.863	26.342	6.253	0.000783	0.002695	15
221222_s_at	NM_017860	C1orf56	chromosome 1 open reading frame 56	66.731	69.355	30.676	0.000795	0.002725	1
221527_s_at	AF196185	PARD3	par-3 partitioning defective 3 homolog (C. elegans)	54.122	22.186	23.536	0.000798	0.002733	10
214109_at	AI659561	LRBA	LPS-responsive vesicle trafficking, beach and anchor containing	194.61	142.08	80.826	0.000805	0.002753	4
210829_s_at	AF077048	SSBP2	single-stranded DNA binding protein 2	72.652	35.295	34.41	0.000805	0.002753	5
210640_s_at	U63917	GPER	G protein-coupled estrogen receptor 1	28.298	22.502	55.401	0.000806	0.002754	7
205919_at	NM_005330	HBE1	hemoglobin, epsilon 1	179.211	125.943	86.953	0.00084	0.002846	11
202815_s_at	NM_006460	HEXIM1	hexamethylene bis-acetamide inducible 1	94.517	46.824	57.253	0.000848	0.002864	17
208156_x_at	NM_031308	EPPK1	epiplakin 1	196.677	383.059	150.198	0.00086	0.00290	8

							4	8	
210935_s_at	AF274954	WDR1	WD repeat domain 1	282.597	580.584	372.648	0.000869	0.00292	4
211136_s_at	BC004865	CLPTM1	cleft lip and palate associated transmembrane protein 1	86.288	108.551	184.543	0.000894	0.002986	19
204432_at	NM_006943	SOX12	SRY (sex determining region Y)-box 12	31.238	14.782	26.862	0.000904	0.003009	20
207156_at	NM_021064	HIST1H2AG	histone cluster 1, H2ag	130.112	106.846	51.834	0.000906	0.003015	6
208396_s_at	NM_005019	PDE1A	phosphodiesterase 1A, calmodulin-dependent	77.107	49.302	34.26	0.000925	0.003061	2
219874_at	NM_024628	SLC12A8	solute carrier family 12 (potassium/chloride transporters), member 8	11.649	12.809	35.868	0.000933	0.003082	3
215058_at	AU144041	MGC24039	hypothetical protein MGC24039	64.612	22.304	36.682	0.000946	0.003114	12
213006_at	AV655640	CEBPD	CCAAT/enhancer binding protein (C/EBP), delta	28.431	9.83	7.358	0.000958	0.003141	8
217523_at	AV700298	CD44	CD44 molecule (Indian blood group)	13.701	23.531	10.613	0.000967	0.003163	11
208216_at	NM_001934	DLX4	distal-less homeobox 4	25.935	13.668	29.322	0.000975	0.003183	17
219529_at	NM_004669	CLIC3	chloride intracellular channel 3	80.502	104.838	38.223	0.000981	0.003197	9
217775_s_at	AF167438	RDH11	retinol dehydrogenase 11 (all-trans/9-cis/11-cis)	442.743	218.839	307.186	0.001022	0.003297	14
201733_at	AA902971	CLCN3	chloride channel 3	62.9	30.615	58.295	0.001024	0.003302	4
206928_at	NM_003431	ZNF124	zinc finger protein 124	257.637	157.658	126.12	0.001052	0.003375	1
219833_s_at	NM_018100	EFHC1	EF-hand domain (C-terminal) containing 1	22.319	18.461	10.686	0.001056	0.003384	6
205351_at	NM_000821	GGCX	gamma-glutamyl carboxylase	11.264	16.189	31.483	0.00109	0.003468	2
209962_at	M34986	EPOR	erythropoietin receptor	26.4	7.16	12.336	0.001104	0.0035	19
221920_s_at	BE677761	SLC25A37	solute carrier family 25, member 37	15.238	35.92	18.277	0.00111	0.003515	8
204646_at	NM_000110	DPYD	dihydropyrimidine dehydrogenase	201.054	156.691	99.268	0.001121	0.003543	1
216071_x_at	AF132033	MED12	mediator complex subunit 12	33.647	25.708	52.081	0.001197	0.003732	X

219517_at	NM_025165	ELL3	elongation factor RNA polymerase II-like 3	77.576	34.477	60.088	0.001253	0.003868	15
219284_at	NM_024610	HSPBAP1	HSPB (heat shock 27kDa) associated protein 1	131.905	50.495	78.547	0.00127	0.003905	3
203788_s_at	AI962897	SEMA3C	sema domain, immunoglobulin domain (Ig), short basic domain, secreted, (semaphorin) 3C	8.903	25.01	12.688	0.0013	0.003983	7
201313_at	NM_001975	ENO2	enolase 2 (gamma, neuronal)	38.126	22.844	49.322	0.001329	0.004045	12
209941_at	U50062	RIPK1	receptor (TNFRSF)-interacting serine-threonine kinase 1	59.518	82.067	35.718	0.001333	0.004058	6
220449_at	NM_024049	MGC5566	hypothetical protein MGC5566	13.01	30.558	35	0.001407	0.00423	20
213158_at	BG251521			136.558	61.179	63.432	0.001425	0.004267	
210233_at	AF167343	IL1RAP	interleukin 1 receptor accessory protein	85.268	212.374	133.976	0.001453	0.004334	3
214683_s_at	AI251890	CLK1	CDC-like kinase 1	161.419	112.998	80.664	0.001501	0.004445	2
219763_at	NM_024820	DENND1A	DENN/MADD domain containing 1A	98.703	148.212	235.916	0.001582	0.004642	9
209060_x_at	AI438999	NCOA3	nuclear receptor coactivator 3	304.853	132.407	179.146	0.001584	0.004647	20
218817_at	NM_021928	SPCS3	signal peptidase complex subunit 3 homolog (S. cerevisiae)	46.923	106.621	109.665	0.00164	0.004776	4
209032_s_at	AF132811	CADM1	cell adhesion molecule 1	69.272	52.83	106.102	0.001688	0.004898	11
219189_at	NM_024555	FBXL6	F-box and leucine-rich repeat protein 6	12.169	13.963	24.932	0.001709	0.004954	8
204109_s_at	BF445142	NFYA	nuclear transcription factor Y, alpha	12.586	28.512	16.776	0.001723	0.004984	6
213156_at	BG251521			44.714	14.187	18.321	0.001736	0.005013	
209468_at	AB017498	LRP5	low density lipoprotein receptor-related protein 5	48.45	36.138	81.139	0.001805	0.005193	11
206272_at	NM_006542	SPHAR	S-phase response (cyclin-related)	97.999	125.302	57.87	0.001811	0.005207	1
218972_at	NM_018259	TTC17	tetratricopeptide repeat domain 17	90.648	69.171	44.31	0.001899	0.005389	11
221989_at	AW057781	RPL10	ribosomal protein L10	53.411	113.309	100.612	0.00191	0.00542	X

							8	7	
219066_at	NM_021823	PPCDC	phosphopantothenoylcysteine decarboxylase	38.694	58.634	80.594	0.002002	0.005613	15
201167_x_at	NM_004309	ARHGDIA	Rho GDP dissociation inhibitor (GDI) alpha	58.327	85.897	133.448	0.002015	0.005642	17
215832_x_at	AV722190	PICALM	phosphatidylinositol binding clathrin assembly protein	213.738	431.076	283.196	0.002021	0.005651	11
212092_at	BE858180	PEG10	paternally expressed 10	22.237	8.707	16.206	0.0021	0.005823	7
205796_at	NM_018393	TCP11L1	t-complex 11 (mouse)-like 1	7.556	21.501	21.867	0.002162	0.005954	11
201107_s_at	AI812030	THBS1	thrombospondin 1	5.387	33.444	5.343	0.002166	0.005962	15
201008_s_at	NM_006472	TXNIP	thioredoxin interacting protein	48.73	25.686	55.967	0.002189	0.006015	1
220770_s_at	NM_022090	LOC63920	transposon-derived Buster3 transposase-like	23.973	11.555	11.13	0.002193	0.006019	5
204863_s_at	BE856546	IL6ST	interleukin 6 signal transducer (gp130, oncostatin M receptor)	139.553	113.505	61.01	0.002207	0.006044	5
213352_at	AB018322	TMCC1	transmembrane and coiled-coil domain family 1	18.802	24.048	11.273	0.002215	0.006053	3
213606_s_at	AI571798	ARHGDIA	Rho GDP dissociation inhibitor (GDI) alpha	71.524	136.98	194.365	0.002236	0.006097	17
204206_at	NM_020310	MNT	MAX binding protein	172.599	86.001	154.201	0.002282	0.006197	17
52005_at	AA422049	WIZ	widely interspaced zinc finger motifs	17.488	22.462	37.927	0.002306	0.006241	19
202685_s_at	AI467916	AXL	AXL receptor tyrosine kinase	40.218	58.522	86.374	0.002393	0.006426	19
210664_s_at	AF021834	TFPI	tissue factor pathway inhibitor (lipoprotein-associated coagulation inhibitor)	18.996	27.884	41.605	0.002445	0.006539	2
209258_s_at	AI373676	SMC3	structural maintenance of chromosomes 3	848.741	416.812	607.093	0.002554	0.006764	10
200637_s_at	AI762627	PTPRF	protein tyrosine phosphatase, receptor type, F	25.194	37.085	55.082	0.002583	0.006828	1
216636_at	AL050043			45.656	42.068	19.641	0.002622	0.006914	
218485_s_at	BC001427	SLC35C1	solute carrier family 35, member C1	14.85	23.973	34.92	0.002633	0.006931	11
222040_at	AI144007	LOC728844	hypothetical LOC728844	32.146	74.16	48.555	0.002639	0.00694	12

212844_at	AI400490	RRP1B	ribosomal RNA processing 1 homolog B (S. cerevisiae)	23.237	40.519	49.325	0.002803	0.007298	21
218791_s_at	NM_024713	C15orf29	chromosome 15 open reading frame 29	40.097	63.313	30.871	0.002931	0.007582	15
219973_at	NM_024590	ARSJ	arylsulfatase family, member J	23.903	42.472	20.695	0.003054	0.007827	4
220020_at	NM_022098	XPNPEP3	X-prolyl aminopeptidase (aminopeptidase P) 3, putative	43.138	25.851	67.134	0.003216	0.008141	22
207510_at	NM_000710	BDKRB1	bradykinin receptor B1	5.06	14.081	5.047	0.003281	0.008272	14
207717_s_at	NM_004572	PKP2	plakophilin 2	189.336	89.686	96.068	0.00341	0.008529	12
205383_s_at	NM_015642	ZBTB20	zinc finger and BTB domain containing 20	261.145	108.193	124.811	0.003501	0.008721	3
215984_s_at	AL121845	ARFRP1	ADP-ribosylation factor related protein 1	45.522	93.694	94.89	0.003522	0.008761	20
220327_at	NM_016206	VGLL3	vestigial like 3 (Drosophila)	66.711	47.857	30.54	0.003564	0.008828	3
208798_x_at	AF204231	GOLGA8A	golgi autoantigen, golgin subfamily a, 8A	20.072	32.168	69.636	0.003686	0.009051	15
203961_at	AL157398	NEBL	nebulette	14.801	12.459	6.491	0.003702	0.00908	10
221919_at	AW450929	LOC728844	hypothetical LOC728844	39.353	81.349	81.254	0.003716	0.009105	12
47553_at	AA813332	DFNB31	deafness, autosomal recessive 31	20.694	49.157	22.402	0.00377	0.009216	9
217707_x_at	AI535683	SMARCA2	SWI/SNF related, matrix associated, actin dependent regulator of chromatin, subfamily a, member 2	156.476	90.548	186.665	0.003812	0.009293	9
221765_at	AI378044	UGCG	UDP-glucose ceramide glucosyltransferase	302.425	189.95	139.272	0.00412	0.009913	9
